# Evidence of hybridization between genetically distinct Baltic cod stocks during peak population abundance(s)

**DOI:** 10.1111/eva.13575

**Published:** 2023-07-06

**Authors:** Cecilia Helmerson, Peggy Weist, Marine Servane Ono Brieuc, Marius F. Maurstad, Franziska Maria Schade, Jan Dierking, Christoph Petereit, Halvor Knutsen, Julian Metcalfe, David Righton, Carl André, Uwe Krumme, Sissel Jentoft, Reinhold Hanel

**Affiliations:** ^1^ Centre for Ecological and Evolutionary Synthesis Department of Biosciences University of Oslo Oslo Norway; ^2^ Thünen Institute of Fisheries Ecology Bremerhaven Germany; ^3^ Institute of Marine Research Bergen Norway; ^4^ Thünen Institute of Baltic Sea Fisheries Rostock Germany; ^5^ GEOMAR Helmholtz Centre for Ocean Research Kiel Germany; ^6^ Gemeinschaftsschule an der Schlei Kappeln Germany; ^7^ Centre for Coastal Research University of Agder Kristiansand Norway; ^8^ Centre for Environment Fisheries and Aquaculture Science Lowestoft UK; ^9^ Department of Marine Sciences – Tjärnö University of Gothenburg Strömstad Sweden

**Keywords:** Baltic Sea, contact zone, *Gadus morhua*, hybridization, inversions, population genetics

## Abstract

Range expansions can lead to increased contact of divergent populations, thus increasing the potential of hybridization events. Whether viable hybrids are produced will most likely depend on the level of genomic divergence and associated genomic incompatibilities between the different entities as well as environmental conditions. By taking advantage of historical Baltic cod (*Gadus morhua*) otolith samples combined with genotyping and whole genome sequencing, we here investigate the genetic impact of the increased spawning stock biomass of the eastern Baltic cod stock in the mid 1980s. The eastern Baltic cod is genetically highly differentiated from the adjacent western Baltic cod and locally adapted to the brackish environmental conditions in the deeper Eastern basins of the Baltic Sea unsuitable for its marine counterparts. Our genotyping results show an increased proportion of eastern Baltic cod in western Baltic areas (Mecklenburg Bay and Arkona Basin)—indicative of a range expansion westwards—during the peak population abundance in the 1980s. Additionally, we detect high frequencies of potential hybrids (including F1, F2 and backcrosses), verified by whole genome sequencing data for a subset of individuals. Analysis of mitochondrial genomes further indicates directional gene flow from eastern Baltic cod males to western Baltic cod females. Our findings unravel that increased overlap in distribution can promote hybridization between highly divergent populations and that the hybrids can be viable and survive under specific and favourable environmental conditions. However, the observed hybridization had seemingly no long‐lasting impact on the continuous separation and genetic differentiation between the unique Baltic cod stocks.

## 1 INTRODUCTION

Hybridization, i.e. interbreeding, between species or genetically divergent populations (jointly referred to as “divergent entities” in the following) is a well‐known biological phenomenon (Arnold, 1997; Macholán, 2013). The impact of hybridization will rely on several factors, including the genomic divergence as well as ecological selection on the parental populations or species (Pekkala et al., 2012; Sambatti et al., 2008). Hybridization often results in individuals with lower survival due to hybrid incompatibilities (postzygotic barriers) caused by deleterious genetic interactions between the two parental genomes (Presgraves, 2010; Pryke & Griffith, 2009; Rogers & Bernatchez, 2006). Lower fitness in intermediate phenotypes compared to their parental phenotypes is also sometimes reported (Hermansen et al., 2011). In some systems, however, hybridization, i.e. genetic exchange between divergent entities, is demonstrated to be globally neutral or beneficial. The maintenance of novel genetic variation (or introgressed regions) will by large depend on its adaptive significance, that is whether these regions include adaptive alleles resulting in phenotypes that are more advantageous than the parental phenotypes (Giska et al., 2019; Norris et al., 2015). Additionally, introgressive hybridization may increase the standing genetic variation (Glasheen et al., 2020; Ronco et al., 2021) and give rise to a higher degree of adaptability and evolutionary potential (Grant & Grant, 2019; Meier et al., 2017), and thus be crucial for overcoming environmental stressors, including those related to ongoing climate change.

In the wild, hybrid zones are established where closely related species or divergent populations of a species overlap in geographical distribution and hybridize (Barton & Hewitt, 1985; Johannesson et al., 2020; Nielsen et al., 2004). Hybrid zones can be formed through either (i) secondary contact after previous separation, where the divergent entities have diverged (by genetic drift or selection) during the period of separation, or via (ii) primary contact where the initial divergence emerge from a common background and is caused by varying selection regimes across an environmental gradient (Endler, 1977; Mayr, 1942). Alterations in environmental conditions can lead to shifts of geographical distribution ranges, and loss of ecological and geographical barriers that have kept closely related species or divergent populations apart, and thus facilitate new contact zones and hybridization events (Brennan et al., 2014; Chunco, 2014; Taylor et al., 2015). Hybrid zones have also been described to be dynamic and more prone to change over time and space than previously thought (Buggs, 2007; Krosby & Rohwer, 2010; Wielstra, 2019). However, the long‐term evolutionary consequences of such contemporary hybridization events directed by environmental changes are hard to predict. For instance, if these events will nurture climatic adaptations of critically endangered and affected populations, likely depend on whether or not the selection regimes favor the hybrids over the parental phenotypes.

The Baltic Sea is a relatively young brackish intercontinental sea, dating back to approx. 8000 BP, and comprises a series of relatively shallow basins formed as the ice retreated at the end of the last ice age (12,600 BP) (Björck, 1995; Leppäranta & Myrberg, 2009). During the transformation, the region proceeded from being fully freshwater to become connected with the marine environment in Kattegat and Skagerrak, through a few narrow straits (Björck, 1995; Leppäranta & Myrberg, 2009), and was colonized by numerous marine species. Most of these species are found in the outer more saline regions (Bonsdorff, 2006). However, some species have successfully colonized also the less saline regions and basins, where signs of local adaptation and thus, changes in important phenotypic traits are recorded (Johansson et al., 2017; Kautsky et al., 1990), including salinity tolerance (Johansson et al., 2017; Renborg et al., 2014; Wood et al., 2014). For most of these species, a genetic cline is observed following the decreasing salinities into the Baltic Sea from the marine conditions in the Skagerrak via the transition zone in the Kattegat and Belt Sea towards the lower saline basins in the Baltic Sea, i.e. Bornholm Basin and Gotland Deep, indicating low or no gene flow between the locally adapted populations (Johannesson et al., 2020; Le Moan et al., 2019; Nielsen et al., 2004). One of the ecologically and economically important marine species that has successfully colonized the Baltic Sea is the Atlantic cod (*Gadus morhua* Linnaeus, 1758). Numerous of reports have documented on the drastic phenotypic modifications that the eastern Baltic cod have undergone, such as changes in egg buoyancy, sperm motility, general osmoregulation as well as spawning depth and season, enabling it to successfully reproduce and cope with the challenges living in a dynamic brackish‐water environment (Nissling et al., 1994; Nissling & Westin, 1997; Petereit et al., 2014). Differences between the eastern and western Baltic cod include a higher salinity requirement for activation of spermatozoa in the western Baltic cod, ranging from 15‰ to 16‰ as opposed to 11‰ to 12‰ for eastern Baltic cod (Nissling & Westin, 1997). Egg buoyancy is also different between the two, with western Baltic reaching neutral buoyancy at 20‰–22 ‰ and eastern at 13.3‰–15.7 ‰ (Nissling et al., 1994; Nissling & Westin, 1997). Differentiation in adaptation to salinity has also been documented for other life stages, including juvenile and adult individuals (Kijewska et al., 2016; Malachowicz & Wenne, 2019). Moreover, the eastern Baltic cod is found to be highly divergent at the genome‐wide level from its marine counterparts including western Baltic cod (Barth et al., 2019; Berg et al., 2015; Poćwierz‐Kotus et al., 2015; Wenne et al., 2020) and is one of several examples of potential ongoing speciation in the Baltic Sea (Johannesson et al., 2020; Momigliano et al., 2017). It should also be noted that two of the four larger chromosomal inversions identified in Atlantic cod which discriminate between populations throughout its geographical distribution (Berg et al., 2016, 2017; Sodeland et al., 2016), are seemingly under strong selection in the Baltic Sea (Berg et al., 2015). The inversion on linkage group (LG) 2 seems to be linked to osmoregulation and oxygen adaptation, while the inversion on LG12 seems to be linked to temperature adaptation (Berg et al., 2015). Such larger chromosomal inversions—or supergenes—are likely to facilitate the observed divergence between the locally adapted populations, and thus, hindering gene flow over the contact zone (Barth et al., 2017, 2019; Johannesson et al., 2020).

**FIGURE 1:**
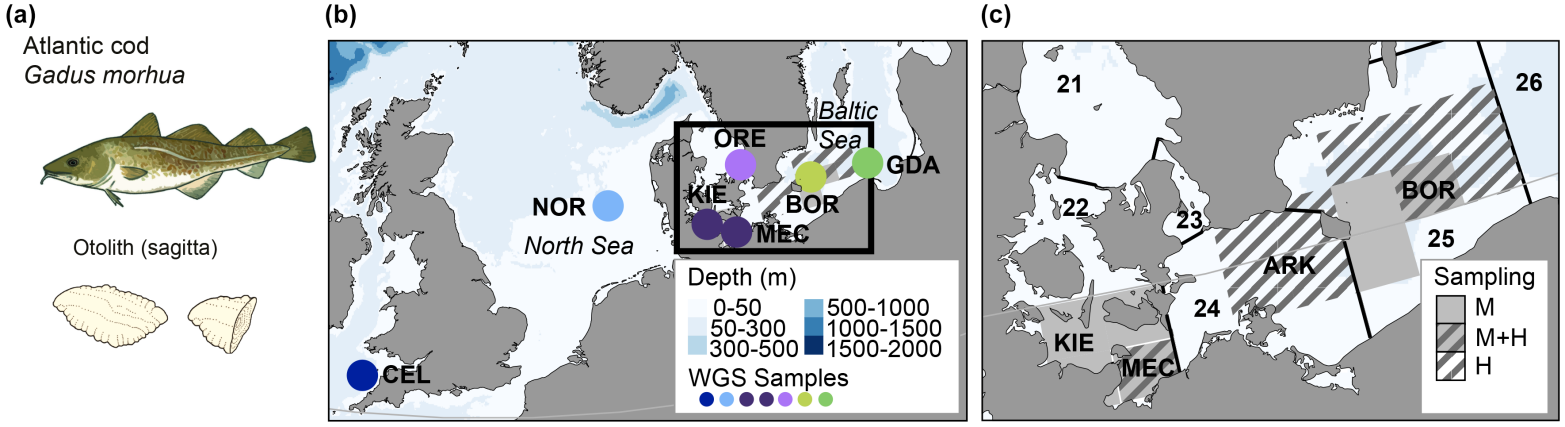
(a) Atlantic cod and sagittal otolith (whole and broken). Illustration: Cecilia Helmerson. Otoliths were used for genetic material and provision of age information. (b) Sampling sites in the Western Atlantic for individuals used in the WGS data‐set (displayed with dots): Celtic Sea (CEL), North Sea (NOR), the transition area into the Baltic Sea, including Oresund (ORE), Kiel Bight (KIE), Mecklenburg Bight (MEC), as well as Bornholm Basin (BOR) and Gdansk Deep (GDA). (c) Sampling areas of the southern part of the Baltic Sea for the individuals genotyped with the diagnostic SNP‐panel (displayed as rectangles): SDs are marked with bold lines and map show SD 22–25 and partially SD 21 and 26. Striped rectangles marking historical sampling sites (H) (1979–1989), grey rectangles marking modern (M) (2016) sampling areas. Grey and striped showing areas with both modern and historical samples (M + H). KIE = Kiel Bight, MEC = Mecklenburg Bight, ARK = Arkona Basin and BOR = Bornholm Basin.

Today, the two genetically divergent cod populations found in the Baltic region are managed as separate stocks: the western Baltic cod (WBC) mainly inhabiting the shallower western Baltic Sea (ICES, 2019a) (ICES subdivisions (SD) 22–24; see Figure 1b,c), and the eastern Baltic cod (EBC) inhabiting the deeper basins of the eastern Baltic Sea (ICES SD 24–32; see Figure 1b,c). Additionally, the distribution of the two stocks overlaps in the Arkona Sea (ARK; SD 24). From scientific trawl catches the mixing ratios of WBC and EBC in ARK (determined by otolith shape combined with genetics) are relatively stable over time, with about two thirds belonging to EBC and one third to WBC both in the 1980s and in recent years (ICES, 2019a; Schade et al., 2022). It should be noted, that a slight shift in the mixing ratio was seen in the 1990s, where the WBC was dominating (or at least) representing half (50%) of the catches (Schade et al., 2022). The observed mixing ratios in this area could potentially be linked to increases and/or declines in the different stocks during the time period (Hemmer‐Hansen et al., 2019; Schade et al., 2022; Stroganov et al., 2018). Despite this reported co‐existence, there are to date no reports of hybridization (Hemmer‐Hansen et al., 2019; ICES, 2021; Weist et al., 2019). Absence of hybridization could be linked to the non‐overlap in spawning time between the two stocks, as the majority of the eastern Baltic cod spawn during summer, i.e. from June to August, whereas western Baltic cod mainly spawn during spring (February–April) (Bleil et al., 2009; Bleil & Oeberst, 2004; Hüssy, 2011; Tomkiewicz & Köster, 1999; Vitale et al., 2005). However, historically (in the 1960s–1980s) a large proportion of EBC spawned during spring (Baranova et al., 2011). In the 1980s, the EBC stock was one of the most productive cod stocks worldwide with annual landings around 400.000 t (tonnes) at its highest and a spawning stock biomass around 600.000 t (ICES, 2019a). During the same period the growth rate recordings of Baltic cod was increasing and thought to be linked to high food availability (Mion et al., 2021; Svedäng & Hornborg, 2017). The combination of overlapping spawning periods and increased population abundance could have led to potential hybridization events and thus, genetic exchange between the two stocks.

In this study, the overall goal was to identify the genetic impact of the recorded historical peak in spawning stock biomass of EBC during the mid 1980s, i.e. the potential hybridization between the two stocks, by taking advantage of geographically resolved historical otolith archives combined with genotyping (Weist et al., 2019) and whole genome sequencing of both historical and contemporary samples.

## 2 MATERIALS AND METHODS

### 2.1 Sample collection and DNA extraction

Otolith samples with adherent blood and/or tissue (see Figure 1a), from historical collections, were the main source material for this study. The selected samples included both western Baltic cod (WBC) and eastern Baltic cod (EBC) caught during scientific surveys from 1979 to 1989 (Table S1) in the Mecklenburg Bay (MEC; Stock division area (SD) 22), Arkona Basin (ARK; SD 24) and Bornholm Basin (BOR; SD 25) (Figure 1c, maps generated in ggOceanMaps (Vihtakari, 2022)). The MEC samples were collected in January, ARK samples mainly in January and February as well as some in November, and BOR samples in February as well as November–December.

The otoliths had been stored individually in paper envelopes at room temperature until processing. DNA was extracted from the dried tissue and/or blood still attached to the archived otoliths. We used a modified protocol with an overnight incubation step during the initial lysis (Cuveliers et al., 2009; Hutchinson et al., 1999). For each specimen, the tissue from both otoliths was used, no matter if they were whole or broken. In total, sufficient DNA was obtained for genotyping of 436 individuals.

The contemporary specimens used in this study were the same samples as described in Weist et al., 2019. In short, the samples were caught 2016 during scientific surveys in MEC and a few samples from Kiel Bight (KIE), all within SD 22 in February–March, July and in BOR in February–March, May–June, December (see Weist et al., 2019 for more details).

### 2.2 Genotyping of historical and contemporary samples

The historical samples were genotyped with the diagnostic SNP panel (*n* = 20, Table S2) designed to discriminate between WBC and EBC together with 7 SNPs located within inversions on three linkage groups: LG02, LG07 and LG12 (Table S2) according to the procedure described in Weist et al., 2019. The same 27 SNPs were also used for the contemporary 2016 samples mentioned above, already published in Weist et al., 2019.

To detect potentially contaminated DNA extracts, three highly polymorphic microsatellite loci were amplified (GmoC18: Stenvik et al., 2006; Tch11, Tch14: O'Reilly, 2000) in a multiplex PCR reaction using the QIAGEN Multiplex PCR Kit. The PCR reaction volume of 10 μL contained 1 μL of DNA template, 5 μL of QIAGEN Multiplex PCR Master Mix and 0.4 μL of each forward and reverse primer. PCR consisted of an initial denaturation step of 15 min at 95°C followed by 28 cycles of 30s at 94°C, 3 min at 57°C, 60s at 72°C and a final elongation step of 45 min at 60°C. Amplicons were sized using an ABI 3100 (Applied Biosystems) capillary sequencer and scored using GeneScan Analysis Software. For each locus and individual, the number of alleles was checked to ensure that individuals with no more than two alleles per locus were included in the final dataset for down‐stream analysis.

### 2.3 Population structure analyses and genetic assignment of historical Baltic cod

**FIGURE 2:**
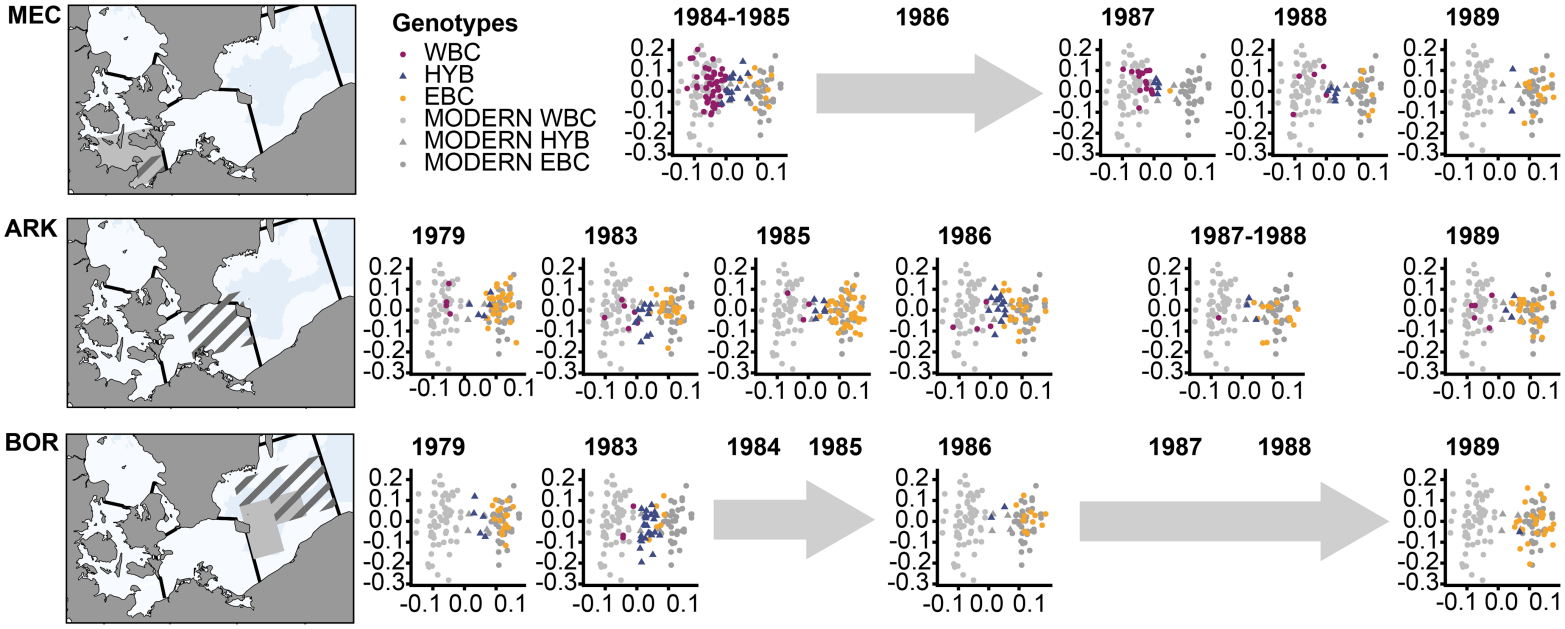
PCA for individual cod in SNP dataset (*N* = 539). PCA is split per sampling location and coloured by genotype. Genotype scoring is divided into WBC (western Baltic cod), HYB (putative hybrids) and EBC (eastern Baltic cod). Modern samples from KIE and BOR used for projection are shown in background regardless of sampling area.

First, assessment of population assignment of EBC and WBC during the early 1980s at the three different locations: MEC, ARK and BOR were analysed with Principal Component Analysis (PCA) using the EIGENSOFT software (SMARTPCA v.6.1.4 and v.7.2.1‐intel‐2018b) (Patterson et al., 2006). To account for high rates of missing genotypes in the historical data, as done previously in datasets with low coverage historical DNA (Star et al., 2017), we calculated the First Principal Component using a set of unambiguously assigned contemporary reference individuals from KIE (*N* = 65; reference to be mostly WBC) and BOR (*N* = 37; reference to be mostly EBC) that had already been genotyped (Weist et al., 2019). Reference individuals are displayed in grey in Figure 2. PCA analyses were conducted by using all 27 SNPs as well as the smaller SNP panel using 19 of the 20 SNPs outside the inversion (Weist et al., 2019). The reduction from 20 to 19 SNPs is based upon STRUCTURE analyses where it was revealed that one of the SNPs in the diagnostic panel had substantial missing data (51.6%), and percentage missing data was further assessed per site in the SNP set with VCFtools 0.1.16 (Table S3).

Second, STRUCTURE (Falush et al., 2003; Pritchard et al., 2000) analyses using the 19 SNPs outside the inversions were performed to further assess the population origin of the historical specimens. The analyses were run with STRUCTURE v. 2.3.4 with FPRIORMEAN set to 0.39 (*F*_ST_ obtained from Weist et al., 2019), with prior population information given for 15 randomly chosen contemporary individuals from BOR and MEC, i.e. belonging to either EBC or WBC respectively. Individuals with an intermediate scoring (0.3–0.7) of EBC or WBC ancestry signal were assigned as putative hybrids (HYB). The selected threshold was chosen to account for some degree of backcrossing as well as hybrids not necessarily scoring at 0.5 (Barth et al., 2020; Elgvin et al., 2017; Zwahlen et al., 2022). Proportions over time in each area (MEC, ARK and BOR) was compared using Fisher's‐exact tests with Bonferroni correction applied.

Third, validation of the 19 SNPs ability to score hybrids was performed by using a modified version of Hybridlab (Nielsen et al., 2006), for more information, see https://github.com/salanova‐elliott/recom‐sim. Theoretical hybrids and backcrosses were obtained using the same 15 predefined EBC and 15 predefined WBC as for the STRUCTURE analyses parents and simulating F1, F2 (first‐ and second‐generation hybrids) as well as B2 and B3 backcrossess (second‐ and third‐generation backcrosses), 100 individuals for each category. The simulated data was then analyzed using STRUCTURE with identical settings as for the real dataset (see above), and the HYB boundaries of 0.3–0.7 were further evaluated.

Additionally, heterozygosity per individual was calculated in VCFtools (using the 20 SNPs), and used for calculation of average heterozygosity for the two populations (EBC and WBC) as well as for the potential hybrids (HYB). These estimations were further used to test if the heterozygosity level was different between the historic and contemporary datasets as well as across cod types in the historical dataset, using two‐way ANOVA, Shapiro–Wilk normality test, Levene's Test for Homogeneity of Variance and Kruskal–Wallis test.

### 2.4 Genome sequencing and hybrid validation

Based upon the STRUCTURE analyses, 11 individuals were chosen for low coverage whole genome sequencing (WGS) to approx. ~0.5‐2X coverage (Table S4). Those selected were either classified as EBC (*N* = 3, *N*_M_ = 2, *N*_F_ = 1, *M* = male, *F* = female), WBC (*N* = 5, *N*_M_ = 5) or potential hybrids (*N* = 3, *N*_M_ = 3). Due to low amount of DNA, the 11 PCR‐free libraries were combined into one common library that underwent a few PCR cycles before sequencing on one lane (using Illumina HiSeq 4000). Sequencing was performed at the Norwegian Sequencing Centre (NSC) at the University of Oslo.

A subset of whole genome sequenced contemporary samples (*N* = 42) were also included. They were all sequenced using Illumina HiSeq 2500 (V4 chemistry, PCR free, 125 bp), some already published (see Barth et al., 2019; marked with*). The contemporary samples (see Figure 1b) were from BOR (BOR 2012*, *N* = 10, *N*_M_ = 5, *N*_F_ = 5), KIE (KIE 2011–2012*, *N* = 10, *N*_M_ = 5, *N*_F_ = 5), the North Sea (NOR) (NOR 2002*, *N* = 5, *N*_M_ = 2, *N*_F_ = 3) and Oresund (ORE) (ORE 2012*, *N* = 10, *N*_M_ = 5, *N*_F_ = 5). In addition, we included samples from the Celtic Sea (CEL) (CEL 2010, *N* = 5, *N*_M_ = 3, *N*_F_ = 2), and Gdańsk Deep (GDA, a part of Baltic Sea) (GDA 2015, *N* = 2, *N*_M_ = na, *N*_F_ = na). See Table S5 for full overview. The samples were chosen randomly from a larger collection (the Aqua Genome project), with an approximately equal number of males and females. The number of contemporary samples were kept on the lower side mainly to not bias the genotyping (i.e. trying also to get a good representation of historically important polymorphic sites), but at the same time with enough power to discriminate between contemporary populations.

The sequencing reads were processed using the Paleomix pipeline (Schubert et al., 2014). Two different forms of BWA (Burrows‐Wheeler Aligner) were used. BWA aln backtrack (Li & Durbin, 2009) (BWA v. 0.5.9‐r26‐dev) was used for historical samples and BWA mem (Li, 2013) for modern (BWA v.0.7.12‐r1039). Both forms were used with alignment to the second version of the cod reference genome: gadMor2 (Tørresen et al., 2016), with the nuclear and the mitochondrial genome given as separate entities. In short, Paleomix pipeline included adapter removal (Lindgreen, 2012) (AdapterRemoval v.2.1.7 for historical and v.1.5 for modern), PCR duplicate removal (Picard‐tools v1.139) http://broadinstitute.github.io/picard/, assessment of MapDamage (Jónsson et al., 2013) (mapDamage v.2.0.6) see Figure S1, alignment (BWA), realignment around indels with GATK (McKenna et al., 2010) (GATK v.3.6‐0‐g89b7209) and filtering of unmapped reads. Subsequently, the bam‐files were further processed following in‐house processing and filtering pipelines (Barth et al., 2019; Star et al., 2017). This included removal of soft‐clipped reads with SAMTOOLS v.1.3.1 (Li et al., 2009). Modern samples were down‐sampled to a coverage of roughly 5X (Samtools v.1.3.1), except for samples that already had 5X coverage or lower (1 sample from BOR and 3 from ORE).

For the genome‐wide detection of SNPs, haplotype and genotype calling was conducted using GATK (v. 3.8) and the gadMor2 genome. Haplotype calling was performed using linear index type and index parameter 128,000. Genotype calling was run using max alternative alleles set as 3. Filtering was performed using BCFtools (v.1.1) and VCFtools (v.0.1.14), starting with expression filtering (BCFtools) using GATK standard (‘FS > 60.0 || MQRankSum<−12.5 || ReadPosRankSum<−8.0 || QD <2.0 || MQ < 40’) and excluding SNPs close to gaps (‐‐SnpGap 10), followed by filtering (VCFtools v.0.1.14) on maximum mean depth (‐‐max‐meanDP 20), removal of indels (‐‐remove‐indels) and only keeping biallelic sites (‐‐min‐alleles 2 –max‐alleles 2). Due to the low coverage of historical samples, filtering was not done by setting a minimum genotype quality, however all DPs under 3 were set as missing (BCFtools v.1.1). After concatenating all linkage groups (BCFtools v.1.1), missingness per site was assessed (VCFtools v.0.1.14) for the historical material and sites identified in more than 5 individuals as missing were removed. It should be noted that a more stringent setting—removing sites with more than 4 individuals missing—was also tested. This filter‐setting, however, reduced the number of sites (*n* = 5259, after pruning for LD) to the point where the separation between modern clusters started to break down (see Figures S2 and S3), so missingness of 5 individuals per site was chosen, which also gave higher resolution with a higher number of SNPs as limit (*n* = 17,359, after pruning for LD). Furthermore, hard to map regions and known repeats were removed (VCFtools v.0.1.14), followed by removal of deaminated sites (awk). For heterozygous sites, we also tested for read depth bias, expecting that true heterozygotes at a site should have an equal probability for each allele. Those sites that fell within an accepted 0.5 frequency using a binominal test using all heterozygote individuals (per site) were kept, whereas those falling outside (*p* < 0.05) were removed, as described in Pinsky et al., 2021. No filtering was made using minimal allele frequency due to small sample sizes. The unplaced scaffolds in the genome were kept throughout all analysis due to the potential of essential genes for Baltic cod not conforming to the reference genome, which is based on the migratory Northeast Arctic cod. Pruning for LD was made using PLINK (v1.90b5.2) with settings –allow‐extra‐chr ‐chr‐set 24 no‐xy ‐indep‐pairwise 10 1 0.8, after removal of inversions at LG02, LG07 and LG12 (Table S6). Ultimately, principal component analysis was performed on the low‐coverage WGS dataset using 53 individuals and 17,359 sites (PLINK v1.90b5.2), using var‐wts 53 (set as number of individuals in PCA). Additional statistics were retrieved running the same dataset through SMARTPCA (EIG v.7.2.1‐intel‐2018b). Fraction explained by each component was calculated in both instances as eigenvalue for the component divided by the sum of the eigenvalues for all components (Figure S4). Heterozygosity was also calculated per individual with VCFtools (0.1.16) in the WGS dataset, for the different cod types. However, further analysis of the heterozygosity was discontinued due to the observed missingness linked to the low coverage in the historical dataset (data not shown).

### 2.5 MT genome analyses

The reads that aligned to mitochondrial (MT) genome—extracted from the WGS dataset using the Paleomix pipeline (see above for details)—were used in haplotype and variant calling (GATK v. 3.8 with the ploidy level defined as haploid). Filtering was made in two steps: GATK standard (‘FS > 60.0 || MQRankSum<−12.5 || ReadPosRankSum<−8.0 || QD <2.0 || MQ < 40’) and excluding SNPs close to gaps (‐‐SnpGap 10), followed by removal of deaminated sites. The final VCF for the MT genomes included 164 SNPs.

Phylogenetic analyses were made in order to look at clustering of maternal linages. To reconstruct the phylogeny of mitochondrial protein coding genes (PCGs), fasta sequences were extracted from the VCF using PPP v0.1.12 (Webb et al., 2021). PCGs were extracted using BLASTN (Camacho et al., 2009), aligned with MAFFT v7.480 (Katoh & Standley, 2013), manually corrected for length, and ND6 was reverse complemented as it is encoded on the light strand. The maximum likelihood analysis, the substitution models, partitioning scheme, and phylogenetic analysis were conducted in IQ‐tree v1.6.12 (Chernomor et al., 2016; Kalyaanamoorthy et al., 2017; Nguyen et al., 2015). Branch support values were obtained using 1000 replicates of ultrafast boostrap (UFBoot) (Hoang et al., 2018), see Figure 5b and Figure S5 (for support values >90). To complement our Maximum likelihood (ML) analysis, a Bayesian phylogeny was inferred using BEAST v2.6.6 (Bouckaert et al., 2019). The PCGs were partitioned into codon positions and the substitution models were inferred using bModelTest (Bouckaert & Drummond, 2017). Analysis was run under the Coalescent Constant Population Tree prior using a strict clock with a chain length of 100,000,000 generations sampling at every 10,000 iterations for a total of 100,000 trees. Convergence was assessed using Tracer 1.7.2 (Rambaut et al., 2018), and a burn in of 10% was selected. TreeAnnotator was used to make a consensus tree (see Figure S6 with posterior probabilites >0.90). Final trees were visualized using the interactive Tree of Life v6.3.1 (iTOL) (Letunic & Bork, 2021).

Phylogenetic analysis was also performed on the complete mitochondrial genome to assess the effect on the topology. For the ML analysis the substitution model was inferred using ModelFinder, before running IQ‐Tree v1.6.12 with 1000 replicates of UFBoot (see Figure S7). Analysis was also run in BEAST v2.6.6 under the Coalescent Constant Population Tree prior using a strict clock with a chain length of 100,000,000 sampling at every 10,000 iterations for a total of 100,000 trees. The substitution model for the Bayesian phylogeny was again inferred using bModelTest. Tracer v1.7.2 was used to assess convergence, and TreeAnnotator with a burn in of 10% was used to create the consensus tree (see Figure S8).

### 2.6 Inversion scoring

The four known inversions in Atlantic cod on LG01, LG02, LG07 and LG12, were scored in the WGS dataset by running local PCAs (with SMARTPCA) using the identified SNPs localized inside the inversions. The inversions were scored either as REF (homozygous for the reference genotype as in gadMor2), HET or NON_REF (homozygous for the non‐reference genotype). The inversion boundaries applied here were already defined in another study with historical samples from the Northeast Arctic ecotype (see Table S6). The inversion on LG02 is defined by two ranges due to a miss‐assembly of the last part of this inversion when comparing to the newest version of the Atlantic cod genome gadMor3 (Matschiner et al., 2022). The number of heterozygous sites using VCFtools v.0.1.14 were calculated at the individual level as in equation:

$$f_{\mathrm{HET}}=\frac{\left(N\_\text{SITES}-O\left(\mathrm{HOM}\right)\right)}{N\_\text{SITES}}$$

Of the total *n* = 17,359 SNPs, 537 SNPs were localized within the inversion on LG01. For LG01 no specific clustering was detected (as expected) since the southernmost Atlantic cod populations are fixed for the non‐reference allele of the inversion on LG01 (Barth et al., 2017; Berg et al., 2015). However, the three latter inversions (on LG02, LG07 and LG12), that have been found to covary in its southernmost distribution (Barth et al., 2017, 2019; Berg et al., 2015; Sodeland et al., 2016, 2022), did display the expected three clusters of the (i) homokaryotypes of the reference genotype, the heterokaryotypes and the homokaryotypes of the alternative genotype arrangement (Mérot, 2020). Within the inversion on LG02, 58 SNPs were identified, whereas 307 and 390 SNPs were identified within the inversions on LG07 and LG12, respectively. In the historical samples, the heterozygote cluster displayed a lower degree of heterozygosity than the contemporary samples (see Figure S9); this was mainly linked to the low coverage combined with a higher degree of missingness per site in the historical dataset (see Figure S10). For the heterozygote cluster on LG07, a tailing pattern was observed, and thus, motivated for further inspection of the genotypes within the inversion (at different filtering steps) using Integrative Genomics Viewer (IGV) v.2.8.9. This investigation resolved 3 out of the 5 individuals with uncertain inversion genotype.

The inversion status for all individuals in the lager historical dataset (*n* = 436) was also investigated using the 7 SNPs localized within the inversions on LG02, LG07 and LG12 (as described above and see lower part of Table S2). First, we did an assessment of the scoring ability of these SNPs (*n* = 2 SNPs on LG02, *n* = 3 SNPs on LG07 and *n* = 2 on LG12). This was done by comparing the results with the scoring conducted on the historical whole genome sequenced individuals (Table S7). Error rate estimations were calculated (not taking missingness into account) as the number of mismatches divided by the total number (of individuals). For further analyses, we selected only the sites with minimal error rate (≤9%). Thus, for LG07 none of the sites were of good quality with error rates ranging from 27% to 45%. For LG02 only one of the sites was selected (at the position 20,868,512), while both sites on LG12 (11,630,885 and 12,529,238) were included (see Table S7). The error rate in the modern WGS dataset was also calculated (Tables S8 and S9), and found to be at a lower level (roughly 4.8%). For LG12, the site with the least missingness in the total historical dataset (see Table S9) at the position 11,630,885 was chosen for the inversion scoring. In the cases of known mismatches scoring from WGS and/or IGV vs. the SNP scoring, the WGS data was more reliable and selected over the SNP scoring.

The inversion genotype frequencies were calculated for the WGS and the SNP genotyped larger historical dataset based on the identified WBC, EBC, and the putative hybrids sampled at the different locations (MEC, ARK and BOR). The inversion genotype frequencies were further tested for HWE (Hardy Weinberg Equilibrium) with the Pearson's Chi‐square test (Pearson, 1900). Differences in inversion genotype frequencies were assessed using Fisher's‐exact test, both between the (i) cod‐types (WBC, EBC, and HYB) and within cod‐types between (ii) the different locations (MEC, ARK and BOR). For the tests conducted within the cod‐types between the different locations, we excluded BOR from the test for the WBC and only tested for differences between MEC and ARK since we here only had three western individuals caught in the BOR area. Correction for multiple testing, i.e. Bonferroni correction, were applied for the Fisher's‐exact tests conducted.

## 3 RESULTS

### 3.1 Population structure and hybrid assessment

Most individuals collected at the different sampling sites (MEC, ARK, BOR) in 1979–1989 and in the contemporary dataset could be assigned genetically as WBC or EBC using PCA, i.e. placed in either of the two clusters shown previously in Weist et al., 2019 (see Figure 2). Furthermore, at all three areas in some years, especially in the mid 1980s, intermediate individuals were detected that neither clustered with the EBC nor the WBC (see Figure 2). The PCA analysis for the SNP set was significant on only the first principal component (Table S10).

**FIGURE 3:**
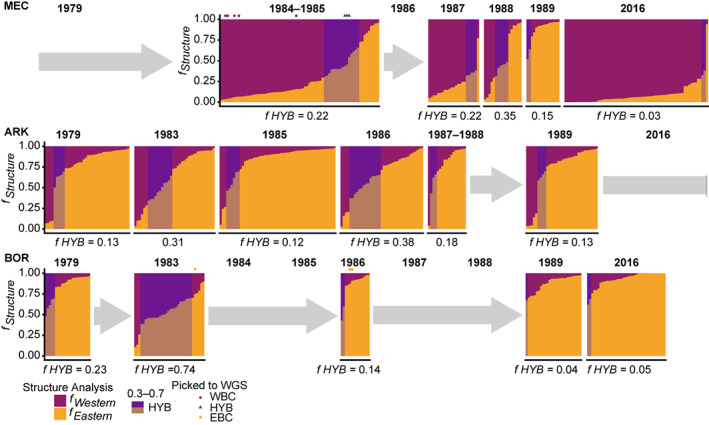
STRUCTURE plots showing fractions (*f*_*Structure*_) for K = 2, split by area (vertical) of Western (purple) and Eastern (orange) genetic signal over time (horizontal). Samples are sorted according to fraction of western signal. Potential hybrids are shadowed as having fractions between 0.3 and 0.7. Samples selected for WGS are marked by purple dot for WBC (western Baltic cod), orange dot for EBC (eastern Baltic cod) and blue triangle for HYB (putative hybrids).

In concordance with the PCA results, STRUCTURE showed a clear separation between WBC (majority purple fraction) and EBC (majority orange fraction), with few individuals showing mixed genetic origin (Figure 3). These individuals were assigned as HYB. Thus, STRUCTURE enabled a final assignment of the individuals as HYB, WBC or EBC (as described above). A higher proportion of HYB individuals were observed in MEC in 1984/1985 (22%), 1987 (22%) and 1988 (35%), in ARK in 1983 (31%) and 1986 (38%), as well as 1983 (74%) for individuals caught in BOR (see Figure 2; Figure S11). It should be noted that even though the classification of the individuals was mostly similar between PCA and STRUCTURE, it did differ for some individuals (Figure 2). Statistically, the genotype proportions (EBC, HYB, WBC) were significantly different over the time period in each area (Table S11). When conducting the pairwise comparisons the statistically differences between timepoints for each area (Tables S12 and S14) could be pinpointed, except for ARK (Table S13).

**FIGURE 4:**
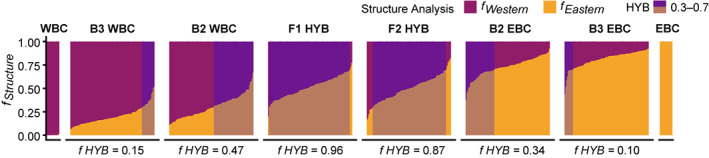
STRUCTURE plots showing fractions (*f*_*Structure*_) for K = 2, of Western (purple) and Eastern (orange) genetic signal for theoretical hybrids and backcrosses as obtained with hybrid simulation tool. WBC is the western parents, B3 is the third‐generation backcrosses, B2 is the second‐generation backcrosses, F1 is the first‐generation hybrids, F2 is the second‐generation hybrids and EBC is the eastern parents. Hybrids within fraction 0.3–0.7 are shadowed and fraction of simulated hybrids picked up given below f _HYB_.

The simulation analysis revealed that the 19 SNPs are powerful enough to detect hybrids and backcrosses (Figure 4). However, simulation show that markers are too few to classify the putative hybrids into pure F1, F2 or backcrosses (fraction levels too similar between the groups). The set hybrid level of 0.3–0.7 in STRUCTURE detects 96% of the simulated F1 hybrids and 87% of the F2 hybrids, as well as 47% of B2 individuals (with WBC as the reference population) and 34% of B2 individuals (with EBC as the refence population), see Figure 4. Thus, the selected hybrid level spanned mainly F1 and F2 but also detected some degree of backcrossing.

The proportions of the two cod types differed at the different locations for specific years. In the contemporary dataset, the majority of individuals caught in MEC—our westernmost sampling site and core area of the WBC population (Hemmer‐Hansen et al., 2019; Weist et al., 2019)—were assigned as WBC (95%), whereas in BOR the majority (95%) of individuals were assigned as EBC. Among the contemporary samples only 4 individuals were scored as hybrids (constituting 3% and 5% of catch 2016 in MEC and BOR respectively).

In our historical dataset, moderate to substantial proportions of the individuals caught in MEC, especially in 1984 and 1985 as well as towards the end of 1980s, were of EBC origin. Especially in the years 1988 and 1989, the individuals caught were assigned as EBC, i.e. 35% and 87%, respectively. Throughout the time period investigated, EBC was dominating the catches in ARK (66%) and BOR (72% when including the samples from 2016).

**FIGURE 5:**
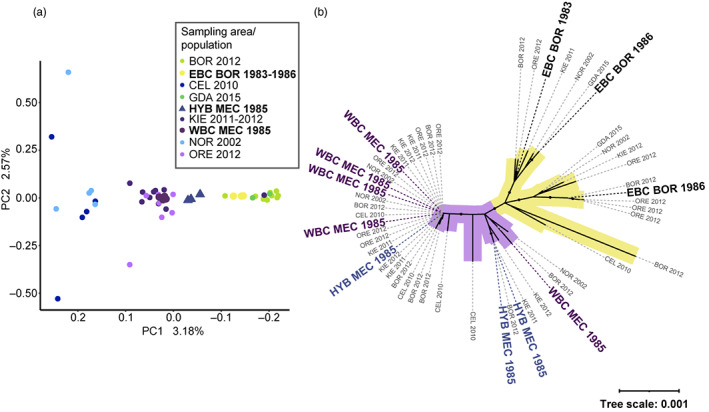
(a) PCA for individuals in WGS dataset (*N* = 53), coloured by sampling area. Point size is larger for historical samples from the SNP set and coloured by assigned population from Structure analysis. (b) Phylogenetic tree based on Protein coding genes (PCGs) Maximum likelihood tree for individuals undergoing WGS (same individuals as in PCA). Modern samples have grey text, historical WBC have purple text, historical EBC have black text and hybrids have blue text. Black points on branches represent bootstrap support values >95. Tree branching separating historical WBC and HYB is purple, branch with historical EBC is marked in yellow.

The WGS dataset (the selected historical samples and contemporary WGS samples) was in line with the larger genotyping data‐set, where the PCA analysis showed that the contemporary and historical samples clustered together, separating into EBC and WBC and putative hybrids (Figure 5a). The PCA analysis was only visualized for PC1 versus PC2 since Tracy–Widom statistics revealed that only the first component was significant (Table S15). The historical and contemporary WBC and EBC were separated on the first component axis (Figure 5a), supported by the ANOVA statistics from SMARTPCA (Tables S16 and S17). The historical WBC samples were not significantly different from the modern cod caught in KIE (*p* = 0.96). However, one of the modern KIE individuals was genetically identified as an EBC, based on the PCA (Figure 5a), but not affecting the outcome in the ANOVA.

The historical EBC samples were not significantly different from the contemporary cod caught in BOR, but the results were less clear (*p* = 0.10), possibly due to the low sample size (*N* = 3). However, the slight off placement of the historical EBC closer to the “putative” hybrids could be indicative of some divergence in relation to the contemporary EBC (BOR). Furthermore, the historical EBC and WBC clusters were defined as significantly different (*p* = 0.00), in line with the significant difference between the contemporary cod caught in KIE and the contemporary cod caught in BOR (*p* = 0.00). The hybrids were significantly different from both historical groups (*p* = 0.00 versus WBC and *p* = 0.04 versus EBC). The two modern GDA samples clustered together with the BOR samples (*p* = 0.73). ORE clustered with KIE (*p* = 0.71). The samples from CEL and NOR clustered together (*p* = 0.50), and separately from all other samples (*p* = 0.00). The Chi‐square statistics—when looking at all eigenvectors together—generally supported the results from the first component (Table S18). None of the 10 sites identified as driving the PC1 of the WGS in SMARTPCA (eigbestsnp) were the same as the sites in the diagnostic SNP panel (Tables S2 and S19). The eigbest were chosen by SMARTPCA, and all had snpweights >4.64 on PC1. Among the eigbestsnp sites only 6 sites were located on the same linkage groups as the sites in the diagnostic SNP panel. The site closest to a SNP in the panel was located 37,841 bp downstream. All the 10 eigbestsnps were located within 2 kb of coding regions (Table S20), 8 were located within introns, one was located between two genes and one was located outside a gene.

**FIGURE 6:**
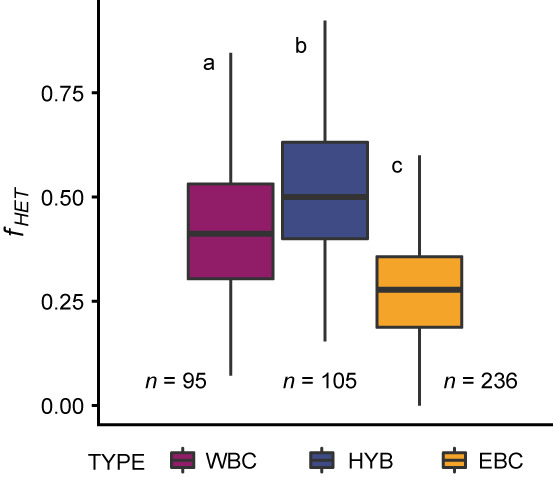
Fractions of heterozygosity (f_HET_) in SNP dataset (*N* = 436, 20 SNPs). Plots coloured by cod type scoring. Significantly different groups are marked with a,b and c.

Measurements of heterozygosity levels using the 20 SNP dataset showed no significant difference between the historical and contemporary dataset nor the interaction between cod type (WBC, HYB, EBC) x dataset (i.e. historical/modern), using two‐way ANOVA (*p* = 0.34 and *p* = 0.31, Table S21, Figures S12 and S13). However, a significant difference between the cod types (*p* < 2e‐16) was detected. We could only test for differentiation in the historical samples due to a failure of parametric test criteria (Shapiro–Wilk normality test of residuals W = 0.99413, *p* = 0.04 and Levene's Test for Homogeneity of Variance, *F* = 5.9526, df = 5, *p* = 2.27e‐05), most likely due to the skewness in numbers of individuals and residuals between historical and contemporary samples (Figure S14). The Kruskal–Wallis test for the historical samples gave a significant difference in heterozygosity level between genotypes (Chi‐square = 134.73, df = 2, *p* < 2.2e‐16), with all genotypes being significantly different from each other, i.e. the EBC vs. WBC vs. hybrids, with the highest heterozygosity level found for the hybrids (Figure 6; Table S22).

### 3.2 Inversion frequencies

**FIGURE 7:**
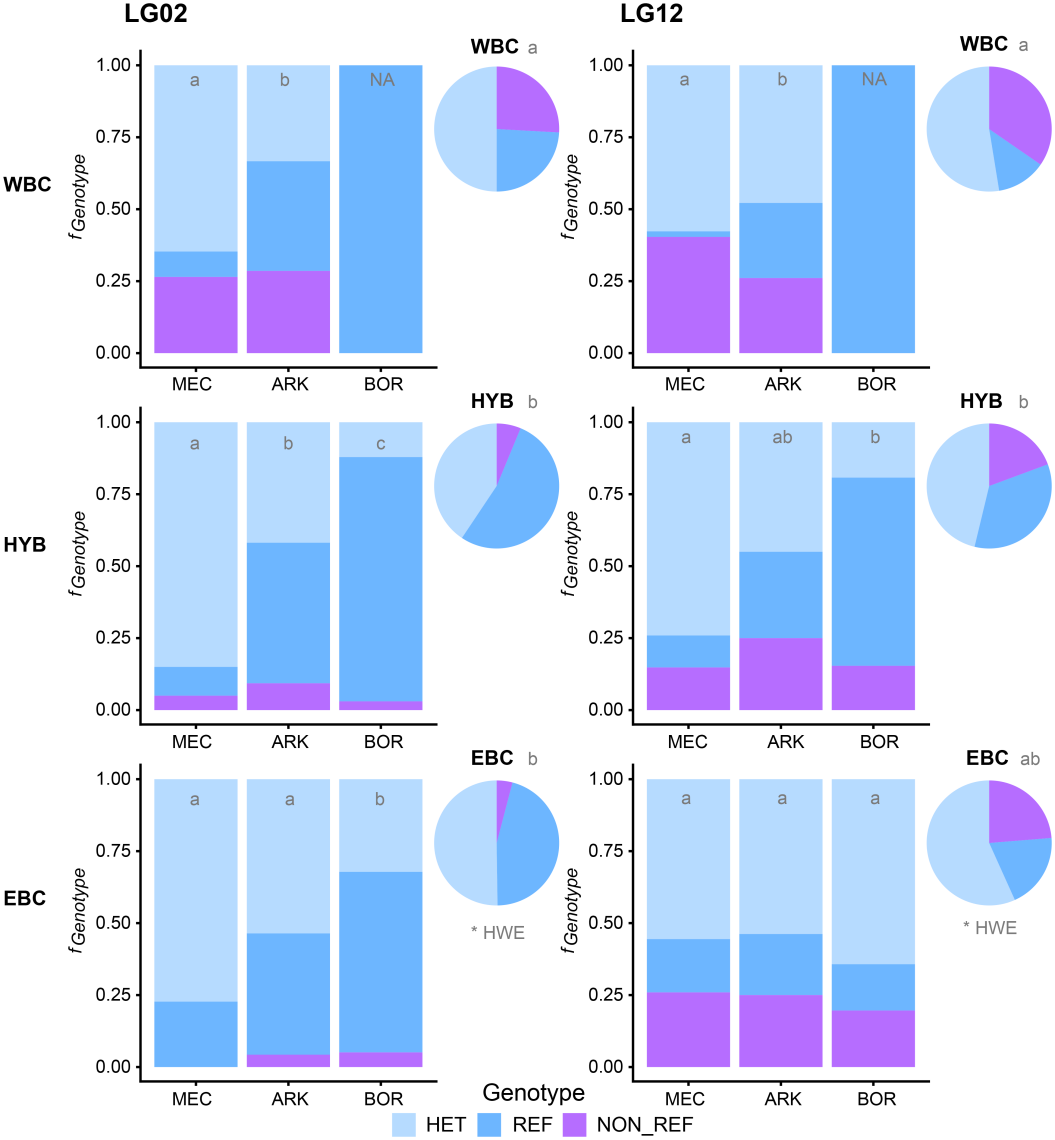
Inversion proportions (f_Genotype_) for inversions at LG02 and LG12, for all historical samples. Proportions split per cod genotype (WBC, HYB, EBC) (pie‐charts) and genotype+sampling area (bar‐plots). Statistical groups are marked with a,b,c. Groups not significantly different are marked with the same letters. NA indicating groups not tested, due to few observations.

Calculation of the inversion frequencies on LG02 and LG12 for the two cod types (EBC and WBC) as well as in the HYB individuals at the different locations (MEC, ARK and BOR) indicates an east–west differentiation for LG02 in all cod types whereas for LG12 only for WBC and HYB (see Figure 7; Tables S23–S25). Almost all comparisons of inversion frequencies on LG02 were significant (Table S24), with the exception of the eastern Baltic MEC‐ARK comparison (*p* = 0.1171). The number of WBC individuals caught at BOR were considered too low for reliable testing (*n* = 3 individuals) even if statistically possible to do. However, it is worth mentioning that all three individuals caught were homozygous for the REF variant on LG02. For all cod types, there was a tendency of an increased number of the LG02 REF variant with cod caught further east (see Figure 7; Table S24).

For LG12, WBC displayed significant differences in inversion frequencies between MEC and ARK (*p* = 5.97e‐03), with an increase in the number of individuals displaying the REF variant vs the NON_REF variant. Again, the number of WBC caught in BOR (*n* = 3) was considered too low for further testing, but all three were homozygous for the REF variant on LG12. For the HYB individuals, even if not that clear since only significantly different between MEC and BOR (*p* = 3.71e‐05), there was a slight but not significant trend for an increasing number for REF variants as seen for WBC. However, for EBC, no significant difference was found between locations (*p* = 0.78) on LG12.

When testing for difference in inversion frequencies between the cod types irrespective of location, we found for LG02 significant differences that were pinpointed to be between the WBC and HYB (*p* = 1.26e‐04) as well as between WBC and EBC (*p* = 2.23e‐06), whereas no difference was found between EBC and HYB (*p* = 0.23). The HYB and EBC individuals displayed a higher frequency of the REF variant than the WBC (see pie‐charts in Figure 7; Tables S26 and S27). For LG12, the results were not as clear‐cut, with WBC deviating from HYB (*p* = 2.09e‐03), but not from EBC (*p* = 0.13), and HYB not being significantly different from EBC (*p* = 0.02). However, in the latter case there might be a trend towards a difference in inversion frequencies on LG12 between EBC and HYB, with HYB displaying a higher number of the REF variant (as also seen for the WBC). When testing inversion frequencies for HWE for the different cod types, only the inversions in EBC were found to be in disequilibrium, both on LG02 and LG12 (Figure 7; Tables S28 and S29). Additionally, we plotted the inversion frequencies for the low coverage WGS dataset (see Figure S15), but due to the low number of historical individuals, no statistical testing was conducted.

### 3.3 Geneflow and demographic inference by MT genome analyses

The mitochondrial analysis displayed two major branches (see Figure 5b), with very little genetic variation within as well as between the clusters. Contemporary samples from both the WBC and EBC were found in both branches together with samples from the Oresund, North Sea and Celtic Sea (all contemporary samples coloured in grey). However, all the historical WBC samples (coloured purple) were in one of the branches together with the potential HYB (blue‐green), whereas the historical EBC specimens assembled in the other branch (yellow). The fact that all HYB individuals clustered with the historical WBC suggests directional gene flow from EBC males to WBC females.

## 4 DISCUSSION

### 4.1 General findings

Genotyping of historical Baltic cod otolith samples uncovered an unexpected large fraction of adult EBC in Mecklenburg Bay and Arkona Basin during the times of peak spawning stock biomass of EBC observed in the 1980s (ICES, 2022), indicative of a range expansion of EBC into western regions. Additionally, a rather high frequency of potential hybrids (including F1, F2 and backcrosses) coincided with this range expansion. Inferring the data from the whole genome sequencing of mitochondrial DNA further indicates directional gene flow from EBC males to WBC females. Taken together, our results demonstrate that hybridization between EBC and WBC has occurred, and is most likely linked to the increase in population abundance combined with overlapping spawning periods.

### 4.2 Range expansion of EBC coinciding with increased spawning stock biomass

Using a selected number of SNPs shown to discriminate between WBC and EBC (Weist et al., 2019) we assigned historical samples of Baltic cod as either belonging to WBC, EBC or being putative hybrids. Intriguingly, the genotyping uncovered that a majority of the individuals caught in our most western Baltic station (MEC, SD22) in the mid 1980s were assigned as EBC. A high proportion of EBC was detected in 1984 and 1985, but also observed towards the end of 1980s (1988 and 1989). This is the first genetic evidence of a regular occurrence of adult (and mature) EBC this far west, in higher saline environmental conditions in historical times. There are, however, numerous of reports documenting the coexistence of the two stocks in the Arkona Basin (SD24), where the mixing ratio seems to be relatively stable, but to some degree impacted by population stock dynamics (Hemmer‐Hansen et al., 2019; Hüssy, 2011; Hüssy et al., 2016; ICES, 2019b; Schade et al., 2022). Our data confirm that the Arkona Basin is by large occupied by EBC in a relatively stable manner during the time period investigated.

It should be noted, that the historical cod specimens in this study were not collected during the main spawning time. For MEC and ARK, however, the fish was caught in January and February just a little ahead of the peak of the spawning time. Moreover, most of the cod caught in MEC were mature (94% had the scoring of maturation stage V and VII), and thus, very likely to spawn in the area they were caught. Consequently, our results suggest that the peak population abundance of EBC (ICES, 2022) could be linked to a range expansion westwards in the 1980s. The massive increase of the EBC spawning stock biomass has been linked to favourable environmental conditions in the late 1970s, with increased inflow of more saline waters from Kattegat into the central and eastern Baltic basins, as well as coupled to the “reactivation” of the spawning areas in the eastern part of the Baltic, such as the Gotland Basin and Gdańsk Deep (Karaseva, 2018; Plikshs et al., 1993). On the other hand, throughout the same time period, only a very minor proportion of the cod caught in the BOR was assigned as WBC. These results suggest that even the more saline and oxygenated conditions in the 1980s did not result in an eastward range expansion of WBC into the lower saline (and deeper) central and eastern Baltic Sea areas. Our results are in line with reviews on the migratory behavior of adult WBC, and are seemingly found at lower frequencies in this area (Hüssy, 2011; Hüssy et al., 2020; Schade et al., 2022).

Recently, Schade et al., 2022 showed that the mixing proportion of WBC and EBC in ARK is linked to water depth, i.e. WBC inhabits more shallower waters whereas EBC is more often found in the deeper waters all year round. Since our historical samples are caught with trawl gear in deeper areas of the basins, the proportion of the WBC vs. EBC observed in this study is not necessary reflecting the real proportion of the two stock types. However, our finding of a westward range expansion of EBC is unlikely to be by chance since in MEC trawls usually operate in relatively shallow waters of 20–25 m depth and samples originated from the pre‐spawning time of WBC when WBC concentrates in those depths.

### 4.3 Evidence of hybridization between the divergent EBC and WBC populations

Due to the well documented coexistence of EBC and WBC mainly in the Arkona Basin, the potential impact on recruitment and/or gene flow between the two stocks have been debated (Heidemann et al., 2012; Hüssy et al., 2016). However, so far, no evidence of hybridization events has been reported (Hemmer‐Hansen et al., 2019). Intriguingly, our genotyping results of the historical samples of Atlantic cod caught at the three different areas, MEC, ARK and BOR, uncovered putative hybrids. These individuals had an intermediate scoring in both the PCA and STRUCTURE analyses, and displayed a higher level of heterozygosity in the smaller SNP panel, indicative of being hybrids. Our results were further supported by whole genome sequencing of a sub‐set of individuals, showing that the putative hybrids were placed as “intermediate” between the EBC and WBC individuals in the PCA (Figure 5a). It should, however, be noted that the historical EBC samples clustered a bit off their contemporary counterparts, and closer to the “putative” hybrids. This could indicate that the EBC has undergone strong selection (perhaps due to e.g. fisheries induced evolution) during the past decades, resulting in an even stronger contemporary genetic separation and divergence of the EBC from the WBC. Alternatively, it could indicate that hybridization was more frequent historically than nowadays. However, caution should be taken due to the small sample sizes as well as low genome sequencing coverage (1X) of these individuals.

The highest proportions of hybrid individuals were uncovered in MEC and ARK, and by large in the mid 1980s. However, a small fraction of hybrids was also detected in the late 1970s in ARK, indicating that the gene flow between the two cod types may have been more frequent in the past. Why these events seem to have taken place in the past while not occurring in modern times could be linked to the more or less overlapping spawning time of the two stocks historically, combined with shifts in environmental conditions and/or variation in spawning stock biomasses. For instance, favourable conditions, with increased inflow of saline waters, have been reported in specific years throughout the 1950s, 1960s and especially in the 1970s (Matthäus & Franck, 1992; Plikshs et al., 1993; Schinke & Matthäus, 1998), which could have led to increased survival of both EBC, WBC and hybrids during those years. In fact, adult hybrids were detected at all main spawning grounds during the late 1970s and mid 1980s, indicating that they survived until maturation and also produced viable offspring, since we see potential second‐generation hybrids (F2) as well as backcrossing (e.g. both B2 and B3 offspring) in our dataset.

In contrast to the high proportion of hybrids present in the past, the contemporary samples show very low numbers (or none in the WGS dataset). The last year with high numbers of hybrids was in 1988 in MEC in our dataset. Our results indicate that the hybridization events between EBC and WBC might be less frequent nowadays (see also Hemmer‐Hansen et al., 2019). It should be noted, as mentioned earlier, that our samples are not likely to display the true fractions of hybrids as well as the two ecotypes, since they are most likely found at different depths and habitats within the different locations. However, the observed decrease in hybrid individuals in modern times could indicate that hybridization may still be occurring but to a lesser extent than historically, due to (i) lower population abundance and maybe most important (ii) the major divergence in spawning time between the two stocks (Hüssy et al., 2016; ICES, 2019b; Köster et al., 2017; Wieland et al., 2000). Abiotic factors are thought to be the major limitation for eastern Baltic cod recruitment (Kosior & Netzel, 1989; Plikshs et al., 1993). Poorer environmental conditions due to, that is, reduced inflow of saline waters during the past decade have resulted in only the southern deeper parts of the Baltic Sea at the halocline (above hypoxic and anoxic depths) available for successful cod spawning and producing vital larvae (Plikshs et al., 1993; Svedäng et al., 2022), and thus could have resulted in lower viability in general including the hybrid individuals.

### 4.4 Inversion genotypes linked to genetic origin and location

We scored the two well‐known inversions found in Atlantic cod of special importance in the Baltic (Barth et al., 2017, 2019; Berg et al., 2015; Matschiner et al., 2022; Sodeland et al., 2016), and inferred the inversion status over the time period and location for the WBC, EBC as well as the hybrids. There was little variation of the inversion frequencies over the time (data not shown), but significant differences in frequencies between WBC and EBC for the inversion on LG02. For LG12 there was no significant difference in frequencies between the two cod stocks. For the hybrids, there seems to be selection against the NON_REF variant, i.e. the REF variant was more frequent as also seen in EBC, at least for the inversion on LG02. It should also be noted that the same tendency—selection against the NON_REF variant—was observed in all cod caught in the Bornholm area irrespective of genetic origin, i.e. assigned as EBC, WBC or hybrids. These results are in line with earlier reports, also demonstrating a higher frequency of the REF variant in the EBC (Barth et al., 2019; Berg et al., 2015). Recently, Matschiner et al., 2022 demonstrated that the REF variant is the ancestral variant of the inversion, whereas the NON_REF variant is the derived variant. Moreover, they also showed that the non‐inverted (REF) inversion genotype has undergone a severe bottleneck in the Baltic cod, further supporting the hypothesis that this variant is of advantage and under selection in the lower saline conditions of the Baltic Sea, possibly also the case for the surviving hybrids caught in this region. A higher frequency of the REF variant is also observed for inversion on the LG12 for the hybrids and WBC. In other words, the potential for favourable allele variants in these inversions is highly likely, and probably more so for the hybrids as well as WBC surviving in the eastern and central Baltic Sea.

### 4.5 MT genome analyses indicate directional gene flow from east to west

Phylogenetic analyses of the MT genome for the whole genome sequencing data‐set, including both historical and contemporary samples, gave further insight into the potential gene flow between the two stocks during the assumed range expansion of EBC in the mid 1980s. The phylogenetic tree separated the samples into two main clusters where the contemporary samples are found mixed in both. The historical samples of WBC and EBC are found in separate clusters, while the hybrids all cluster with the historical WBC samples. This indicates that surviving hybrids had a WBC mother and an EBC father. Caution should however be taken here, due to the low sample size. That said, these results are in concordance with previous phylogenetic analyses, where the overall mitogenomic variation in modern Atlantic cod is demonstrated to be quite high, while the eastern Baltic cod were mainly found in two of the clusters (Jørgensen et al., 2018; Lait et al., 2018; Martínez‐García et al., 2021).

Further evidence for directional gene flow is supported by the phenotypic difference between the two stocks in terms of egg buoyancy and sperm motility (Nissling et al., 1994; Nissling & Westin, 1997; Westin & Nissling, 1991), both important factors for spawning success and survival. WBC is not able to successfully reproduce in the central and eastern Baltic Sea, due to the fact that the eggs (adapted to higher saline environmental conditions) will sink to the deeper layers in the water column with no or low oxygen content and thus fail to develop to hatching (Nissling et al., 1994; Westernhagen, 1970). Experimental studies have demonstrated that sperm activity for EBC is highest between 15.5% and 26‰ salinity, whereas the sperm motility is drastically reduced at lower salinities, which also negatively impact the fertilization (Westin & Nissling, 1991). However, EBC has been shown to spawn sporadically with success in western Baltic areas suggested to be linked to favourable hydrographic conditions and/or spill over from neighbouring spawning areas (Stroganov et al., 2018). Taken this into consideration together with coexistence of the two stocks (in the western Baltic regions in the 1980s) when spawning times were more or less overlapping, could result in hybridization events between the two stocks. Furthermore, hybrids resulting from crosses of female WBC and male EBC would have a higher chance of survival due to the neutral buoyancy of those eggs even in water of lower salinity (Nissling & Westin, 1997). Moreover, mapping of spawning habitat suitability in the western Baltic regions during the mid 1980s to the early 1990s uncovered poor conditions during the main spawning season in January–March, whereas it was considered to be more favourable during the late spawning season in April/May (Hüssy et al., 2012). These findings could indicate a higher survival of eggs and larvae of the late spawners, i.e. such as the EBC (Wieland et al., 2000; Wieland & Horbowa, 1996), and thus, give rise to a higher probability of successful hybridization events during that time period.

## 5 CONCLUDING REMARKS

In this study, we demonstrate how ecosystem alterations, such as increased recruitment and spawning stock biomass of a marine fish stock (due to potential favourable environmental conditions), could mediate (i) range expansion, (ii) increased degree of mixing, and (iii) hybridization between genetically divergent populations.

An increased understanding of such shifts in the ecosystem dynamics and cryptic population division is relevant for stock assessment(s) and can support the development of sustainable management programmes for marine resources (Reiss et al., 2009). For instance, it is likely that many of the tagging experiments and observations conducted in the Baltic region have been confounded by this mixing of the two stocks, which have probably fluctuated with changes in EBC and WBC spawning stock biomass, as indicated by the results in this study and other studies reporting that the population dynamics is a key factor for the degree of mixing of the two stocks (Hemmer‐Hansen et al., 2019; Schade et al., 2022; Stroganov et al., 2018). From a management perspective, it is important to know the extent of hybridization over time as well as degree of physical mixing of divergent populations so that harvesting regimes do not have unintended consequences.

## CONFLICT OF INTEREST STATEMENT

The authors declare no conflicts of interest.

## Supporting information


Data S1.

## Data Availability

All raw sequences from the historical dataset and the modern dataset (those that are not already published) are deposited in the European Nucleotide Archive (ENA) at EMBL‐EBI, project accession number: PRJEB62422. The genotyping data is available on Figshare repository https://doi.org/10.6084/m9.figshare.23014556.
