# Supplementary material for: Evidence of hybridization between genetically distinct Baltic cod stocks during peak population abundance(s)
Source: Evol Appl. 2023 Jul 6;16(7):1359–76. doi: 10.1111/eva.13575 (PMC10363836; doi:10.1111/eva.13575)
Supplement: Supplementary file 1 — Data S1. [file EVA-16-1359-s001.docx]

**Supplementary material**

**Evidence of hybridization between genetically distinct Baltic cod stocks during peak population abundance(s)**

**Cecilia Helmerson^1^, Peggy Weist^2^, Marine Servane Ono Brieuc^1,3^, Marius F. Maurstad^1^, Franziska Maria Schade^4^, Jan Dierking^5^, Christoph Petereit^6^, Halvor Knutsen^3,7^, Julian Metcalfe^8^, David Righton^8^, Carl André^9^, Uwe Krumme^4^, Sissel Jentoft^1*^ and Reinhold Hanel^2*^**

^1^ Centre for Ecological and Evolutionary Synthesis, Department of Biosciences, University of Oslo, Oslo, Norway

^2^ Thünen Institute of Fisheries Ecology, Bremerhaven, Germany

^3^Institute of Marine Research, Bergen, Norway

^4^Thünen Institute of Baltic Sea Fisheries, Rostock, Germany

^5^GEOMAR Helmholtz Centre for Ocean Research Kiel, Germany

^6^Gemeinschaftsschule an der Schlei, Kappeln, Germany

^7^Centre for Coastal Research, University of Agder, Kristiansand, Norway

^8^Centre for Environment Fisheries and Aquaculture Science, Lowestoft, United Kingdom

^9^Department of Marine Sciences – Tjärnö, University of Gothenburg, Strömstad, Sweden

*** Correspondence:**Sissel Jentoft
sissel.jentoft@ibv.uio.no

Reinhold Hanel
reinhold.hanel@thuenen.de

**Table of contents**

[**2** **Materials and Methods** 3](#_Toc135904500)

[**2.1 Sample collection and DNA extraction** 3](#_Toc135904501)

[**2.2 Genotyping of historical and contemporary samples** 5](#_Toc135904502)

[**2.4 Genome sequencing and hybrid validation** 7](#_Toc135904503)

[**2.5 MT genome analyses** 15](#_Toc135904504)

[**2.6 Inversion scoring** 19](#_Toc135904505)

[**3. Results** 24](#_Toc135904506)

[**3.1 Population structure and hybrid assessment** 24](#_Toc135904507)

[**3.2 Inversion frequencies** 39](#_Toc135904508)

[**References** 44](#_Toc135904509)

# **2 Materials and Methods**

#### **2.1 Sample collection and DNA extraction**

**Table S1** Metadata summary for cod samples used for genotyping 436 historical individuals (caught between 1979 and 1989) at 27 SNP loci. Data divided by sampling areas. Sampling month and maturity stage (according to Tomkiewicz et al., 2003) given separately for males and females. Sampling areas stated as MEC (Mecklenburg Bight and neighbouring areas), ARK (Arkona) and BOR (Bornholm) and as SD (stock division) areas - following ICES management areas.

*Continued on next page*

|  | **1979** | **1983** | **1984** | **1985** | **1986** | **1987** | **1988** | **1989** |
| --- | --- | --- | --- | --- | --- | --- | --- | --- |
| **MEC / SD 22** |  |  | 4 | 68 |  | 23 | 17 | 15 |
| **Jan** |  |  | 4 | 68 |  | 23 | 17 | 15 |
| III |  |  |  |  |  |  |  | 7 |
| Females | |  |  |  |  |  |  | 4 |
| Males |  |  |  |  |  |  |  | 3 |
| V |  |  | 4 | 60 |  | 18 | 17 | 8 |
| Females | |  | 4 | 1 |  | 6 |  |  |
| Males |  |  |  | 59 |  | 12 | 17 | 8 |
| VI |  |  |  | 8 |  | 5 |  |  |
| Females | |  |  |  |  | 2 |  |  |
| Males |  |  |  | 8 |  | 3 |  |  |
| **ARK / SD 24** | 38 | 36 |  | 52 | 37 | 5 | 12 | 32 |
| **Jan** |  |  |  | 52 | 13 | 5 |  | 24 |
| II |  |  |  |  |  |  |  | 2 |
| Females | |  |  |  |  |  |  | 2 |
| III |  |  |  |  |  |  |  | 17 |
| Females | |  |  |  |  |  |  | 9 |
| Males |  |  |  |  |  |  |  | 8 |
| IV |  |  |  |  |  |  |  | 1 |
| Males |  |  |  |  |  |  |  | 1 |
| V |  |  |  | 39 | 11 | 5 |  | 4 |
| Males |  |  |  | 39 | 11 | 5 |  | 4 |
| VI |  |  |  | 13 | 2 |  |  |  |
| Males |  |  |  | 13 | 2 |  |  |  |
| **Feb** |  |  |  |  | 4 |  | 12 | 8 |
| III |  |  |  |  |  |  |  | 5 |
| Females | |  |  |  |  |  |  | 2 |
| Males |  |  |  |  |  |  |  | 3 |
| IV |  |  |  |  |  |  |  | 1 |
| Males |  |  |  |  |  |  |  | 1 |
| V |  |  |  |  | 4 |  | 12 | 2 |
| Males |  |  |  |  | 4 |  | 12 | 2 |
| **Nov** | 38 |  |  |  |  |  |  |  |
| I | 5 |  |  |  |  |  |  |  |
| Females | 3 |  |  |  |  |  |  |  |
| Males | 2 |  |  |  |  |  |  |  |
| II | 22 |  |  |  |  |  |  |  |
| Females | 8 |  |  |  |  |  |  |  |
| Males | 14 |  |  |  |  |  |  |  |
| III | 10 |  |  |  |  |  |  |  |
| Females | 7 |  |  |  |  |  |  |  |
| Males | 3 |  |  |  |  |  |  |  |
| IV | 1 |  |  |  |  |  |  |  |
| Females | 1 |  |  |  |  |  |  |  |
| **Dec** |  | 36 |  |  | 20 |  |  |  |
| I |  | 1 |  |  |  |  |  |  |
| Males |  | 1 |  |  |  |  |  |  |
| II |  | 23 |  |  | 14 |  |  |  |
| Females | | 16 |  |  | 7 |  |  |  |
| Males |  | 7 |  |  | 7 |  |  |  |
| III |  | 11 |  |  | 5 |  |  |  |
| Females | | 6 |  |  | 2 |  |  |  |
| Males |  | 5 |  |  | 3 |  |  |  |
| IV |  | 1 |  |  | 1 |  |  |  |
| Males |  | 1 |  |  | 1 |  |  |  |
| **BOR / SD 25** | 22 | 34 |  |  | 14 |  |  | 27 |
| **Feb** |  |  |  |  |  |  |  | 27 |
| I |  |  |  |  |  |  |  | 1 |
| Males |  |  |  |  |  |  |  | 1 |
| II |  |  |  |  |  |  |  | 5 |
| Females | |  |  |  |  |  |  | 3 |
| Males |  |  |  |  |  |  |  | 2 |
| III |  |  |  |  |  |  |  | 12 |
| Females | |  |  |  |  |  |  | 8 |
| Males |  |  |  |  |  |  |  | 4 |
| IV |  |  |  |  |  |  |  | 6 |
| Females | |  |  |  |  |  |  | 2 |
| Males |  |  |  |  |  |  |  | 4 |
| V |  |  |  |  |  |  |  | 3 |
| Males |  |  |  |  |  |  |  | 3 |
| **Nov** | 22 |  |  |  |  |  |  |  |
| II | 15 |  |  |  |  |  |  |  |
| Females | 7 |  |  |  |  |  |  |  |
| Males | 8 |  |  |  |  |  |  |  |
| III | 7 |  |  |  |  |  |  |  |
| Females | 6 |  |  |  |  |  |  |  |
| Males | 1 |  |  |  |  |  |  |  |
| **Dec** |  | 34 |  |  | 14 |  |  |  |
| I |  | 1 |  |  |  |  |  |  |
| Males |  | 1 |  |  |  |  |  |  |
| II |  | 14 |  |  | 10 |  |  |  |
| Females | | 13 |  |  | 5 |  |  |  |
| Males |  | 1 |  |  | 5 |  |  |  |
| III |  | 12 |  |  | 3 |  |  |  |
| Females | | 6 |  |  | 2 |  |  |  |
| Males |  | 6 |  |  | 1 |  |  |  |
| IV |  | 6 |  |  | 1 |  |  |  |
| Males |  | 6 |  |  | 1 |  |  |  |
| V |  | 1 |  |  |  |  |  |  |
| Males |  | 1 |  |  |  |  |  |  |
|  | **1979** | **1983** | **1984** | **1985** | **1986** | **1987** | **1988** | **1989** |

#### **2.2 Genotyping of historical and contemporary samples**

*Continued from previous page*

**Table S2** List of 27 SNP loci used for population analyses (see Weist et al., 2019 for detailed locus information), with marker ID, linkage group and position, both as in gadMor2 genome (Tørresen et al., 2016). Allele frequencies (AF) shown for the contemporary reference individuals (WBC | EBC). Table first listing the 20 SNPs outside the inversions, then the 7 SNPs located within the inversions at LG02, LG07 and LG12.

| ***Marker ID*** | ***Linkage group*** | ***Position*** | ***Description*** | ***AF_x_*** | ***AF_y_*** |
| --- | --- | --- | --- | --- | --- |
| LG01_10417249_SEL | LG01 | 10417249 | not inversion | 0.21 \| 0.77 | 0.79 \| 0.23 |
| LG02_01358822_SEL | LG02 | 1358822 | not inversion | 0.26 \| 0.85 | 0.74 \| 0.15 |
| LG02_14506653_SEL | LG02 | 14506653 | not inversion | 0.2 \| 0.74 | 0.8 \| 0.26 |
| LG03_07053408_SEL | LG03 | 7053408 | not inversion | 0.9 \| 0.38 | 0.1 \| 0.63 |
| LG04_23059256_SEL_60 | LG04 | 23059256 | not inversion | 0.15 \| 0.73 | 0.85 \| 0.27 |
| LG06_00784232_SEL | LG06 | 784232 | not inversion | 0.29 \| 0.83 | 0.71 \| 0.17 |
| LG07_23819790_SEL | LG07 | 23819790 | not inversion | 0.48 \| 0.02 | 0.52 \| 0.98 |
| LG09_08678526_SEL | LG09 | 8678526 | not inversion | 0.71 \| 0.06 | 0.29 \| 0.94 |
| LG09_16657913_SEL | LG09 | 16657913 | not inversion | 0.34 \| 0.17 | 0.66 \| 0.83 |
| LG11_01922930_SEL | LG11 | 1922930 | not inversion | 0.24 \| 0.01 | 0.76 \| 0.99 |
| LG11_20277669_SEL | LG11 | 20277669 | not inversion | 0.68 \| 0.01 | 0.32 \| 0.99 |
| LG12_07553923_SEL | LG12 | 7553923 | not inversion | 0.83 \| 0.21 | 0.17 \| 0.79 |
| LG12_11560045_SEL | LG12 | 11560045 | not inversion | 0.84 \| 0.58 | 0.16 \| 0.42 |
| LG16_22359890_SEL | LG16 | 22359890 | not inversion | 0.15 \| 0 | 0.85 \| 1 |
| LG17_09361714_SEL | LG17 | 9361714 | not inversion | 0.62 \| 0.65 | 0.38 \| 0.35 |
| LG18_04074216_SEL | LG18 | 4074216 | not inversion | 0.4 \| 0.71 | 0.6 \| 0.29 |
| LG18_17089172_SEL | LG18 | 17089172 | not inversion | 0.57 \| 0.96 | 0.43 \| 0.04 |
| LG21_04164158_SEL | LG21 | 4164158 | not inversion | 0.88 \| 0.39 | 0.12 \| 0.61 |
| LG21_18500787_SEL | LG21 | 18500787 | not inversion | 0.23 \| 0.66 | 0.77 \| 0.34 |
| LG22_04333346_SEL | LG22 | 4333346 | not inversion | 0.6 \| 0.24 | 0.4 \| 0.76 |
| LG02_18724285_I02 | LG02 | 18724285 | inversion | 0.29 \| 0.86 | 0.71 \| 0.14 |
| LG02_20868512_I02 | LG02 | 20868512 | inversion | 0.92 \| 0.3 | 0.08 \| 0.7 |
| LG07_14812281_I07 | LG07 | 14812281 | inversion | 0.75 \| 0.54 | 0.25 \| 0.46 |
| LG07_16410308_I07 | LG07 | 16410308 | inversion | 0.46 \| 0.41 | 0.54 \| 0.59 |

**2.3 Population structure analyses and genetic assignment of historical Baltic cod**

**Table S3** Assessment of missing data per site for diagnostic SNPs, listing marker ID, linkage group and position as in gadMor2, and f MISS the fraction of missing data. The site with highest missing data is marked in bold.

| ***Marker ID*** | ***Linkage group*** | ***Position*** | ***f MISS*** |
| --- | --- | --- | --- |
| LG01_10417249_SEL | LG01 | 10417249 | 0.250464 |
| LG02_01358822_SEL | LG02 | 1358822 | 0.0797774 |
| LG02_14506653_SEL | LG02 | 14506653 | 0.133581 |
| **LG03_07053408_SEL** | **LG03** | **7053408** | **0.51577** |
| LG04_23059256_SEL_60 | LG04 | 23059256 | 0.226345 |
| LG06_00784232_SEL | LG06 | 784232 | 0.246753 |
| LG07_23819790_SEL | LG07 | 23819790 | 0.187384 |
| LG09_08678526_SEL | LG09 | 8678526 | 0.111317 |
| LG09_16657913_SEL | LG09 | 16657913 | 0.0946197 |
| LG11_01922930_SEL | LG11 | 1922930 | 0.269017 |
| LG11_20277669_SEL | LG11 | 20277669 | 0.19295 |
| LG12_07553923_SEL | LG12 | 7553923 | 0.107607 |
| LG12_11560045_SEL | LG12 | 11560045 | 0.0816327 |
| LG16_22359890_SEL | LG16 | 22359890 | 0.339518 |
| LG17_09361714_SEL | LG17 | 9361714 | 0.179963 |
| LG18_04074216_SEL | LG18 | 4074216 | 0.118738 |
| LG18_17089172_SEL | LG18 | 17089172 | 0.2282 |
| LG21_04164158_SEL | LG21 | 4164158 | 0.155844 |
| LG21_18500787_SEL | LG21 | 18500787 | 0.124304 |
| LG22_04333346_SEL | LG22 | 4333346 | 0.0705009 |

#### **2.4 Genome sequencing and hybrid validation**

**Table S4** List of 11 individuals chosen for WGS with information about ID (both sample ID and alternative ID), sex, length, age, weight, maturity (scale following Tomkiewicz et al., 2003) and year of catch, as well as STRUCTURE classification and achieved coverage in nuclear and mitochondrial genome in Paleomix (Schubert et al., 2014) for gadMor2 (Tørresen et al., 2016). Fraction of heterozygosity in MT genome calculated from when treating MT genome as diploid, to assess potential contamination issues. The only individuals with excessive heterozygosity are the ones with lowest nuclear coverage (marked in bold), thus not excluded. Furthermore the individuals had previously been checked for contamination with microsatellite loci. Structure classification abbreviated as WBC = western Baltic cod, HYB = hybrid, EBC = eastern Baltic cod. Abbreviation nu = nuclear whereas MT= mitochondrial. f HET = fraction heterozygous sites.

| ***Sample ID*** | ***Alternative ID (Full)*** | ***Sex*** | ***Length***  ***(cm)*** | ***Weight***  ***(kg)*** | ***Maturity*** | ***Age***  ***(years)*** | ***Year of catch*** | ***STRUCTURE classification*** | ***Coverage nu*** | ***Coverage MT*** | ***f HET MT*** |
| --- | --- | --- | --- | --- | --- | --- | --- | --- | --- | --- | --- |
| Sample_73 | Sample_73-VI10B | M | 35.5 | 0.35 | 2 | 2 | 1986 | EBC ⚫ | 2.44 | 54.8 | 0.012 |
| Sample_74 | Sample_74-VI3B | F | 61.5 | 2 | 3 | 7 | 1986 | EBC ⚫ | 1.08 | 94.1 | 0.002 |
| Sample_75 | Sample_75-IV8B | M | 56.5 | NA | 3 | 4 | 1983 | EBC ⚫ | 1.45 | 517.3 | 0 |
| **Sample_76** | **Sample_76-8522-4-19** | **M** | **62.5** | **NA** | **5** | **5** | **1985** | **HYB ▲** | **0.71** | **42.9** | **0.096** |
| Sample_77 | Sample_77-8522-4-25 | M | 51.5 | NA | 5 | 3 | 1985 | WBC ⚫ | 1.61 | 126.8 | 0.005 |
| Sample_78 | Sample_78-8522-4-5 | M | 55.5 | NA | 5 | 4 | 1985 | WBC ⚫ | 0.94 | 84.3 | 0 |
| Sample_79 | Sample_79-8522-16-94 | M | 44.5 | NA | 5 | 3 | 1985 | HYB ▲ | 1.15 | 41.1 | 0.002 |
| **Sample_80** | **Sample_80-8522-4-13** | **M** | **53.5** | **NA** | **6** | **4** | **1985** | **HYB** ▲ | **0.42** | **32.9** | **0.108** |
| Sample_81 | Sample_81-8522-4-54 | M | 64.5 | NA | 5 | 5 | 1985 | WBC ⚫ | 1.45 | 57.4 | 0.002 |
| Sample_82 | Sample_82-8522-4-22 | M | 53.5 | NA | 5 | 4 | 1985 | WBC ⚫ | 1.13 | 48.2 | 0.01 |
| Sample_83 | Sample_83-8522-16-10 | M | 43.5 | 0.85 | 5 | 3 | 1985 | WBC ⚫ | 1.57 | 124.2 | 0 |

**2.4 Genome sequencing and hybrid validation**

**Table S5** List of 42 individuals chosen from the AquaGenome project (CEES, 2022). Information given about ID (both sample ID and alternative ID), sex length, age, weight, maturity (scale following Tomkiewicz et al., 2003) and year of catch, sampling area, as well as nuclear coverage before and after down-sampling. Fraction of heterozygosity in MT genome calculated from when treating MT as diploid, to assess potential contamination issues. Sex noted as, M=Male, F=Female, NA= sex not determined). Sampling area marked with symbols used in PCA analysis.

*Continued on next page*

| ***Sample ID*** | ***Sex*** | ***Length***  ***(cm)*** | ***Weight***  ***(kg)*** | ***Maturity*** | ***Age***  ***(years)*** | ***Year of catch*** | ***Sampling Area*** | ***Cover-age nu*** | ***Coverage nu down*** | ***f HET MT*** |
| --- | --- | --- | --- | --- | --- | --- | --- | --- | --- | --- |
| BOR1205004 | M | 41 | 0.555 | 5 | NA | 2012 | BOR 2012 ⚫ | 7.42 | 4.97 | 0.002 |
| BOR1205005 | M | 42 | 0.632 | 6 | NA | 2012 | BOR 2012 ⚫ | 7.19 | 4.96 | 0 |
| BOR1205007 | M | 45 | 0.754 | 5 | NA | 2012 | BOR 2012 ⚫ | 7.41 | 4.96 | 0 |
| BOR1205008 | M | 46 | 0.826 | 6 | NA | 2012 | BOR 2012 ⚫ | 6.96 | 5.01 | 0 |
| BOR1205009 | F | 38 | 0.625 | 6 | NA | 2012 | BOR 2012 ⚫ | 12.8 | 5 | 0.002 |
| BOR1205010 | F | 47 | 1.131 | 6 | NA | 2012 | BOR 2012 ⚫ | 10.34 | 4.97 | 0 |
| BOR1205013 | F | 52 | 1.465 | 6 | NA | 2012 | BOR 2012 ⚫ | 10.04 | 4.92 | 0 |
| BOR1205014 | F | 38 | 0.54 | 6 | NA | 2012 | BOR 2012 ⚫ | 10.43 | 5.01 | 0 |
| BOR1205015 | F | 36 | 0.584 | 6 | NA | 2012 | BOR 2012 ⚫ | 1.01 | NA | 0 |
| BOR1205017 | M | 31 | 0.227 | 5 | NA | 2012 | BOR 2012 ⚫ | 7.61 | 5.03 | 0.002 |
| CEL1002001 | M | 75 | 5.4 | running | NA | 2010 | CEL 2010 ⚫ | 9.82 | 5.01 | 0.002 |
| CEL1002003 | F | 82 | 7.4 | NA | NA | 2010 | CEL 2010 ⚫ | 8.36 | 4.94 | 0.005 |
| CEL1002013 | M | 69 | 3.8 | running | NA | 2010 | CEL 2010 ⚫ | 7.97 | 4.94 | 0.005 |
| CEL1002014 | M | 79 | 5.2 | running | NA | 2010 | CEL 2010 ⚫ | 9.01 | 4.96 | 0.002 |
| CEL1002022 | F | 95 | 13 | NA | NA | 2010 | CEL 2010 ⚫ | 8.44 | 4.98 | 0 |
| GDA1509004 | NA | 43 | 0.615 | NA | NA | 2015 | GDA 2015 ⚫ | 10.62 | 5.1 | 0 |
| GDA1509005 | NA | 34 | 0.352 | NA | NA | 2015 | GDA 2015 ⚫ | 8.65 | 4.93 | 0 |
| KIE1103004 | F | 37 | NA | 6 | NA | 2011 | KIE 2011-2012 ⚫ | 7.89 | 4.97 | 0 |
| KIE1103013 | F | 39 | NA | 6 | NA | 2011 | KIE 2011-2012 ⚫ | 10.55 | 5.07 | 0.002 |
| ***Sample ID*** | ***Sex*** | ***Length***  ***(cm)*** | ***Weight***  ***(kg)*** | ***Maturity*** | ***Age***  ***(years)*** | ***Year of catch*** | ***Sampling Area*** | ***Cover-age nu*** | ***Coverage nu down*** | ***f HET MT*** |
| KIE1103019 | M | 49 | NA | 6 | NA | 2011 | KIE 2011-2012 ⚫ | 14.84 | 5.05 | 0 |
| KIE1103020 | M | 42 | NA | 6 | NA | 2011 | KIE 2011-2012 ⚫ | 12.87 | 5.02 | 0.002 |
| KIE1103027 | F | 35 | NA | 6 | NA | 2011 | KIE 2011-2012 ⚫ | 10.56 | 5.07 | 0 |
| KIE1103029 | M | 43 | NA | 6 | NA | 2011 | KIE 2011-2012 ⚫ | 7.57 | 5 | 0 |
| KIE1203001 | F | 51 | 1.606 | 6 | NA | 2012 | KIE 2011-2012 ⚫ | 7.99 | 4.95 | 0.002 |
| KIE1203002 | F | 69 | 3.79 | 6 | NA | 2012 | KIE 2011-2012 ⚫ | 8.08 | 5.01 | 0 |
| KIE1203003 | M | 63 | 2.556 | 6 | NA | 2012 | KIE 2011-2012 ⚫ | 8.04 | 4.99 | 0 |
| KIE1203004 | M | 72 | 4.084 | 6 | NA | 2012 | KIE 2011-2012 ⚫ | 7.97 | 4.95 | 0 |
| NOR020302 | F | 64 | 2.67 | 4 | 3 | 2002 | NOR 2002 ⚫ | 8.1 | 5.02 | 0 |
| NOR020313 | M | 61 | 2.594 | 3 | 3 | 2002 | NOR 2002 ⚫ | 8.03 | 4.98 | 0.002 |
| NOR020317 | M | 63 | 2.477 | 4 | 3 | 2002 | NOR 2002 ⚫ | 8.14 | 5.05 | 0.002 |
| NOR020318 | F | 58 | 2.644 | 4 | 3 | 2002 | NOR 2002 ⚫ | 7.76 | 5.04 | 0 |
| NOR020323 | F | 66 | 3.326 | 3 | 4 | 2002 | NOR 2002 ⚫ | 8.12 | 5.04 | 0.002 |
| ORE1203001 | F | 59.9 | 2.032 | Mature | NA | 2012 | ORE 2012 ⚫ | 7.26 | 4.94 | 0.002 |
| ORE1203002 | F | 45.7 | 1.243 | Mature | NA | 2012 | ORE 2012 ⚫ | 5.57 | 4.96 | 0 |
| ORE1203003 | F | 55 | 1.638 | Mature | NA | 2012 | ORE 2012 ⚫ | 6.94 | 5 | 0 |
| ORE1203004 | F | 46.5 | 0.972 | Mature | NA | 2012 | ORE 2012 ⚫ | 4.8 | NA | 0.005 |
| ORE1203005 | M | 54.7 | 1.356 | Mature | NA | 2012 | ORE 2012 ⚫ | 7.48 | 4.94 | 0.005 |
| ORE1203008 | M | 47.5 | 1.056 | Mature | NA | 2012 | ORE 2012 ⚫ | 4.36 | NA | 0 |
| ORE1203009 | M | 54.6 | 1.313 | Mature | NA | 2012 | ORE 2012 ⚫ | 4.1 | NA | 0 |
| ORE1203010 | M | 52.5 | 1.199 | Mature | NA | 2012 | ORE 2012 ⚫ | 8.54 | 4.95 | 0 |
| ORE1203011 | F | 54.7 | 1.557 | Mature | NA | 2012 | ORE 2012 ⚫ | 6.22 | 5.1 | 0.002 |
| ORE1203012 | M | 52.8 | 1.26 | Mature | NA | 2012 | ORE 2012 ⚫ | 5.71 | 5.03 | 0 |

*Continued from previous page*

**2.4 Genome sequencing and hybrid validation**

*
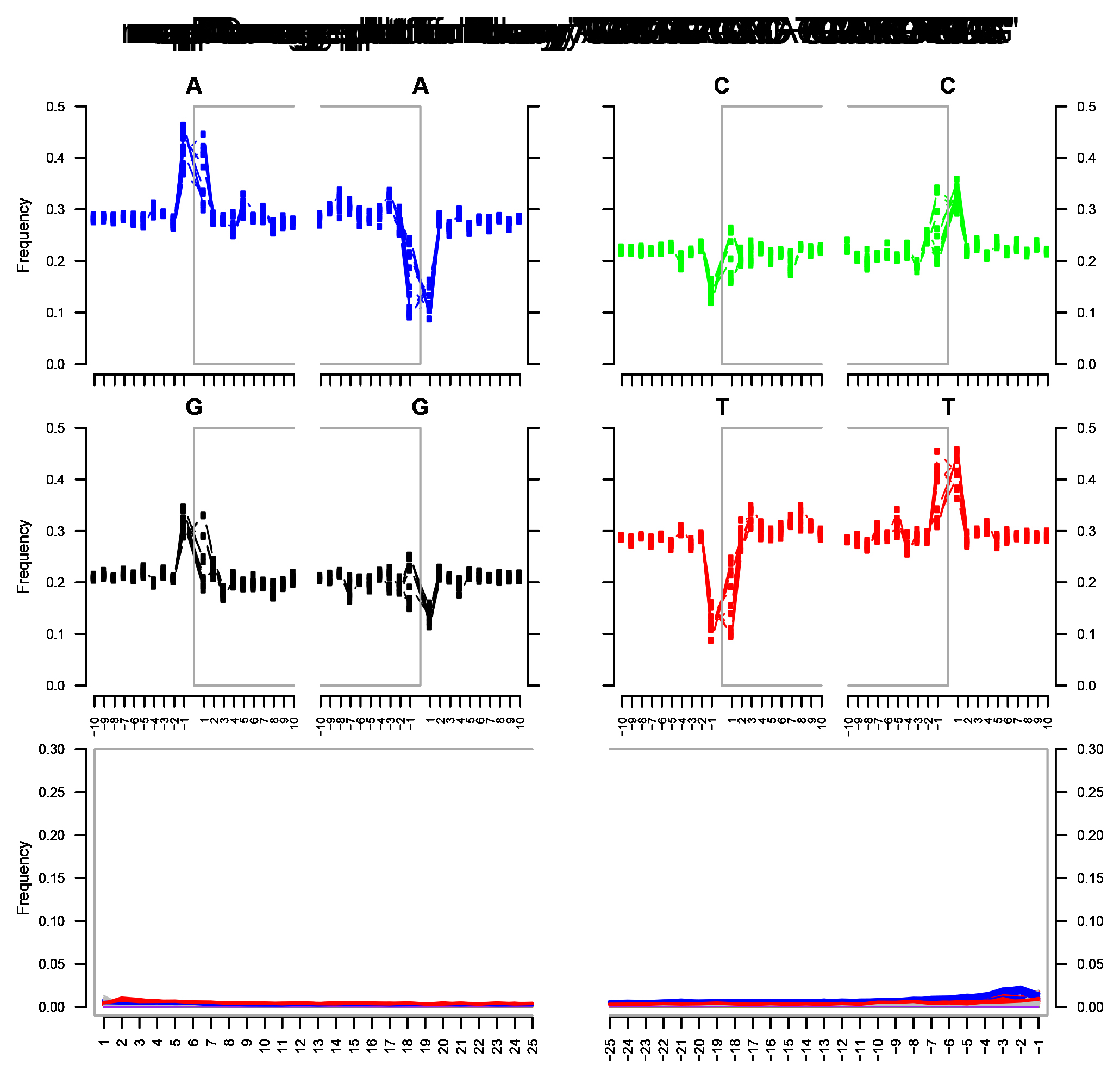
*

**Figure S1** mapDamage (Jónsson et al., 2013) fragmisincorporation plots for 11 historical samples. Position on x-axis, frequency on y-axis. Frequency going from 0-0.5 for top two rows for each sample and from 0-0.3 for last row.

**2.4 Genome sequencing and hybrid validation**


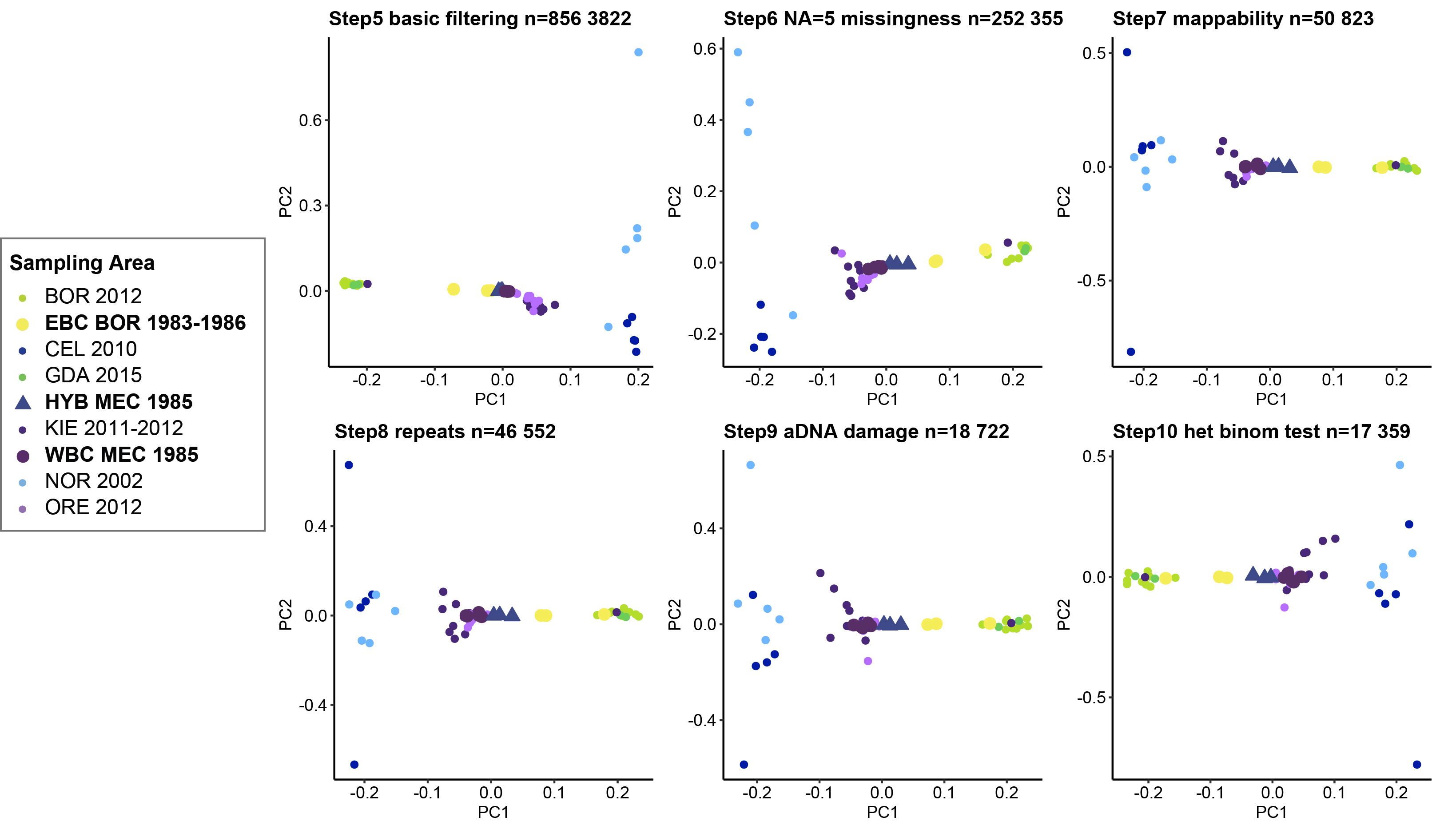


**Figure S2** SMARTPCA plots - filtering step 5 to 10. Individuals coloured by sampling location and year. Number of sites stated (n). Step5 is when all basic filtering has been made, Step6 where filtering is made based on missing data in the historical material 5 individuals allowed to be missing genotype information at sites, Step7 the stage where hard to map regions are excluded and Step8 where known repetitive regions are excluded, Step9 where deaminated sites excluded and Step10 removal of heterozygous sites failing a test of heterozygote allele frequencies.

**2.4 Genome sequencing and hybrid validation**


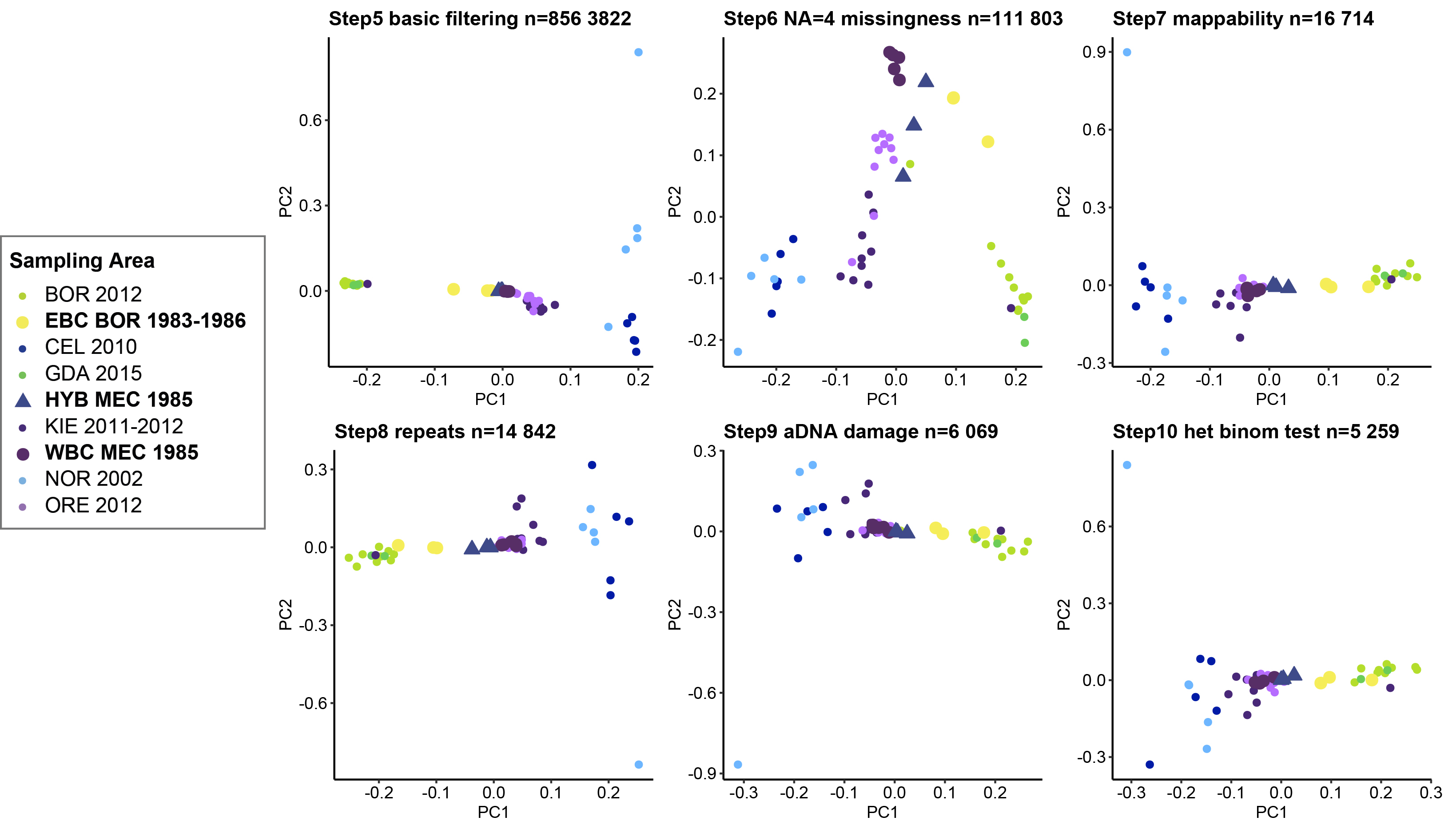


**Figure S3** SMARTPCA plots - filtering step 5 to 10, from step 6 an alternative filtering pipeline. Individuals coloured by sampling location and year. Number of sites stated (n). Step5 is when all basic filtering has been made, Step6 where filtering is made based on missing data in the historical material 4 individuals allowed to be missing genotype information at sites, Step7 the stage where hard to map regions are excluded and Step8 where known repetitive regions are excluded, Step9 where deaminated sites excluded and Step10 removal of heterozygous sites failing a test of heterozygote frequencies.

**2.4 Genome sequencing and hybrid validation**

**Table S6** Borders for inversions as in gadMor2 reference genome when looking within inversions and when excluding inversions. Borders are defined in another study with historical samples from the Northeast Arctic ecotype, using 192 individuals and a dataset with 166 484 sites comparing historical and modern Northeast Arctic cod against modern Norwegian coastal cod and genetical/geographical outgroups from Gulf of Maine, North Sea and Bornholm.

|  | **Chromosome** | **From position** | **To position** |
| --- | --- | --- | --- |
| Within | LG01 | 9165000 | 26130000 |
|  | LG02 | 18555000 | 22035000 |
|  | LG02 | 23220000 | 23969406 |
|  | LG07 | 13665000 | 22965000 |
|  | LG12 | 925001 | 13320000 |
| Excluding | LG01 | 9015000 | 26325000 |
|  | LG02 | 18405000 | 24054406 |
|  | LG07 | 13515000 | 23115000 |
|  | LG12 | 1 | 13920000 |

**2.4 Genome sequencing and hybrid validation**

*
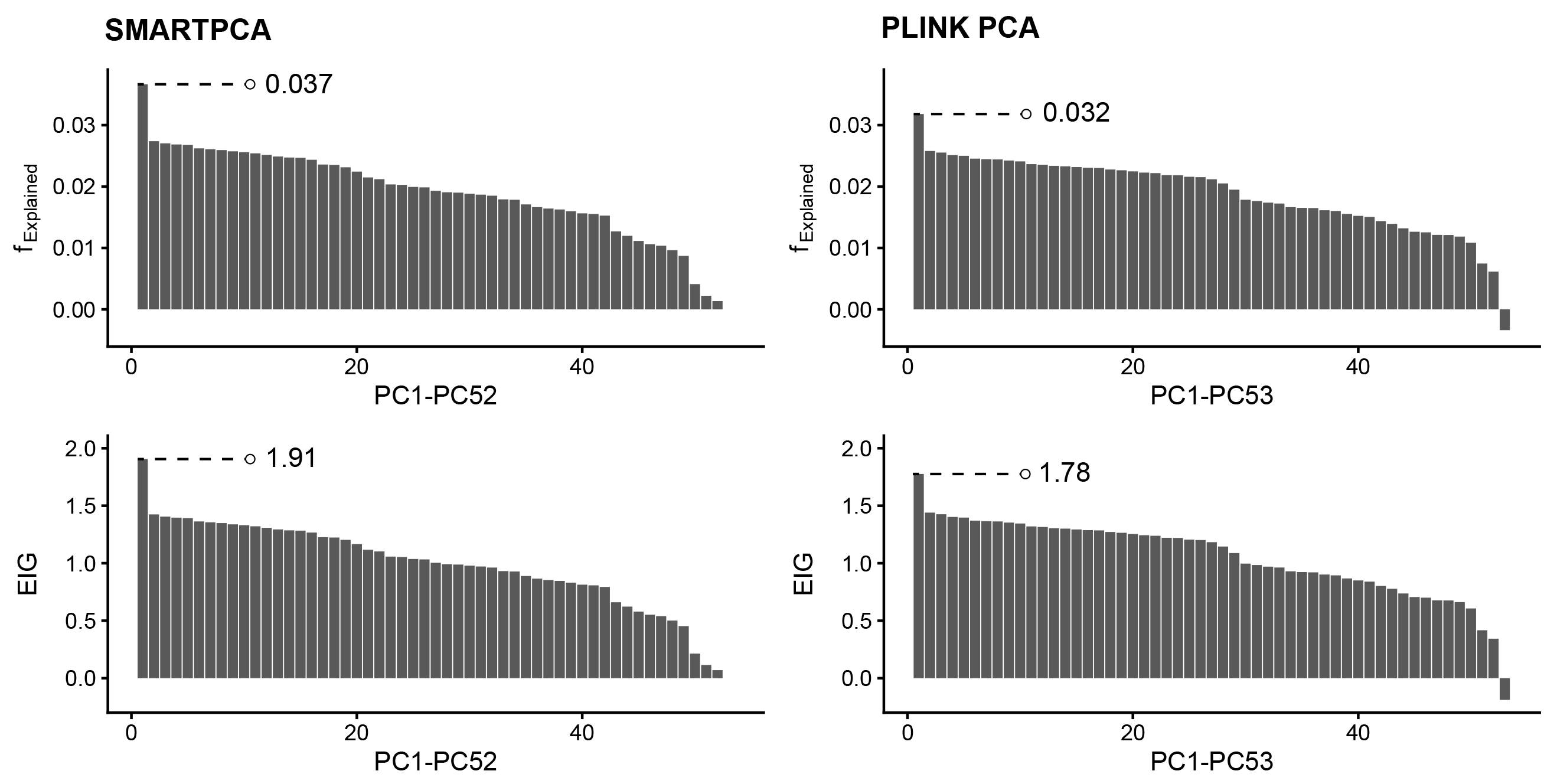
*

**Figure S4** Plot of f Explained (fraction explained) by PCA for WGS dataset, for each axis/component in SMARTPCA (right) and PLINK PCA (left), followed by eigenvalues (bottom) for each programme.

#### **2.5 MT genome analyses**


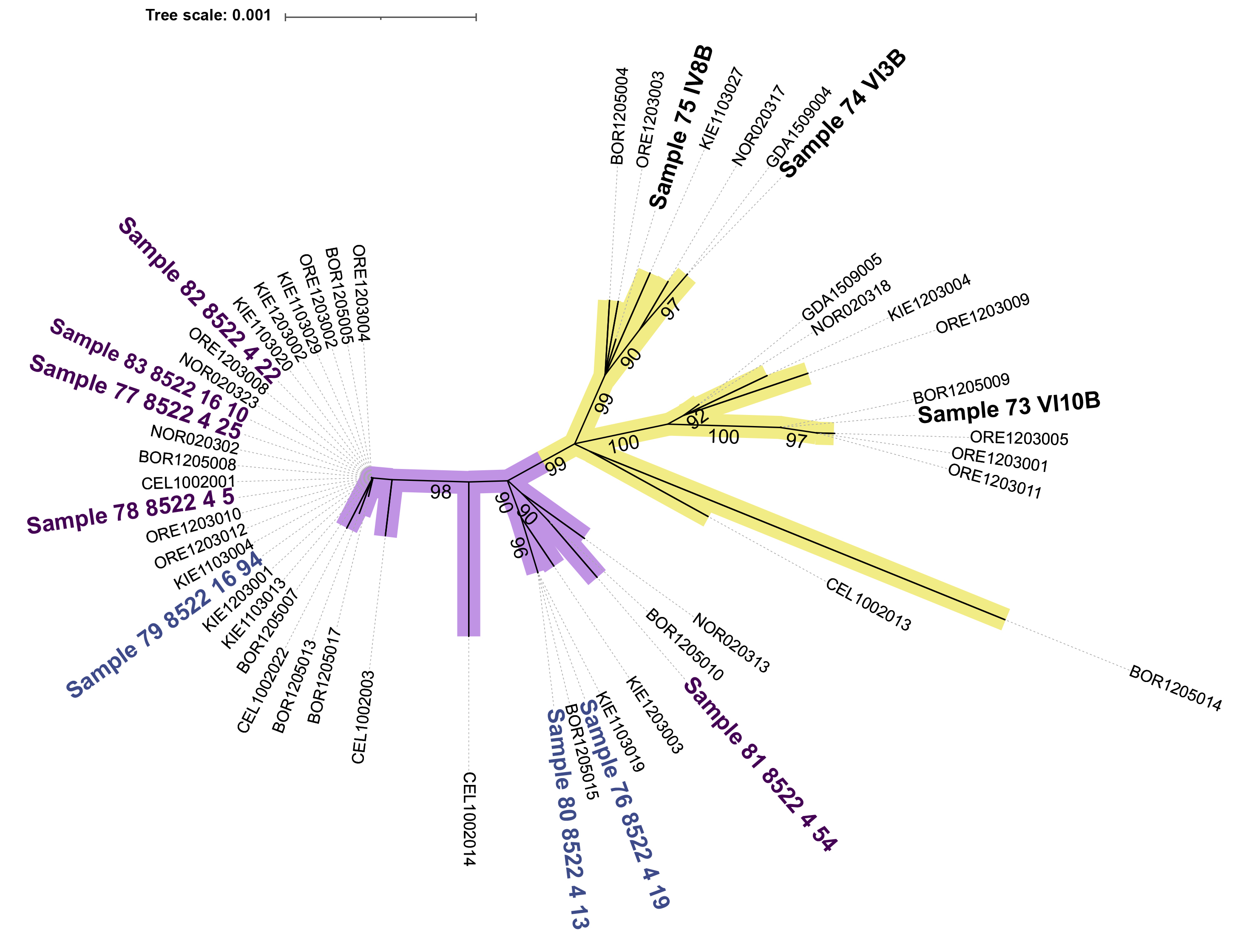


**Figure S5** Phylogenetic tree for mitochondrial protein coding genes, based on maximum likelihood analysis. Support values >90 are shown.

**2.5 MT genome analyses**


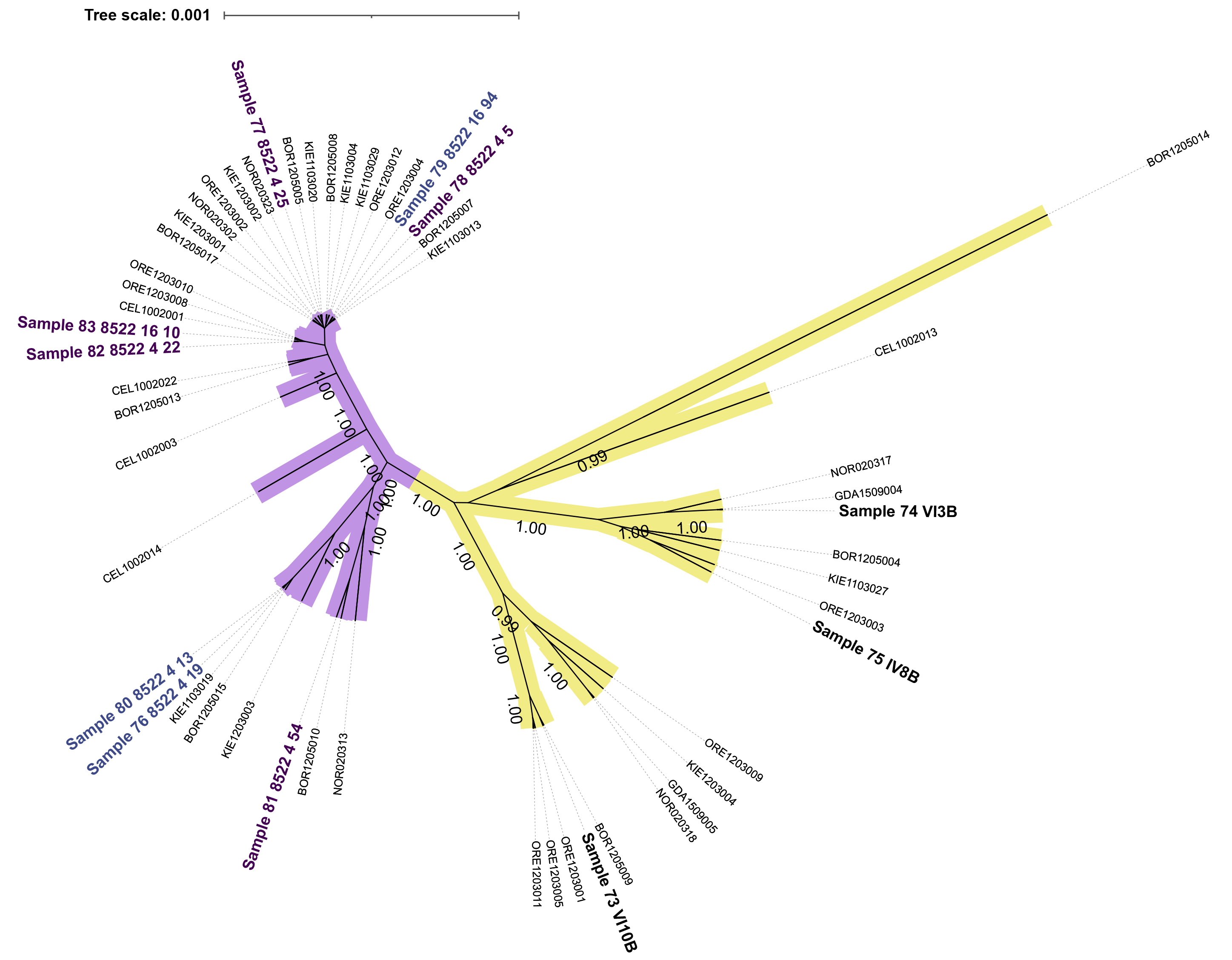


**Figure S6** Phylogenetic tree for mitochondrial protein coding genes, based on Bayesian analysis. Posterior probabilites >0.90 are shown.

**2.5 MT genome analyses**


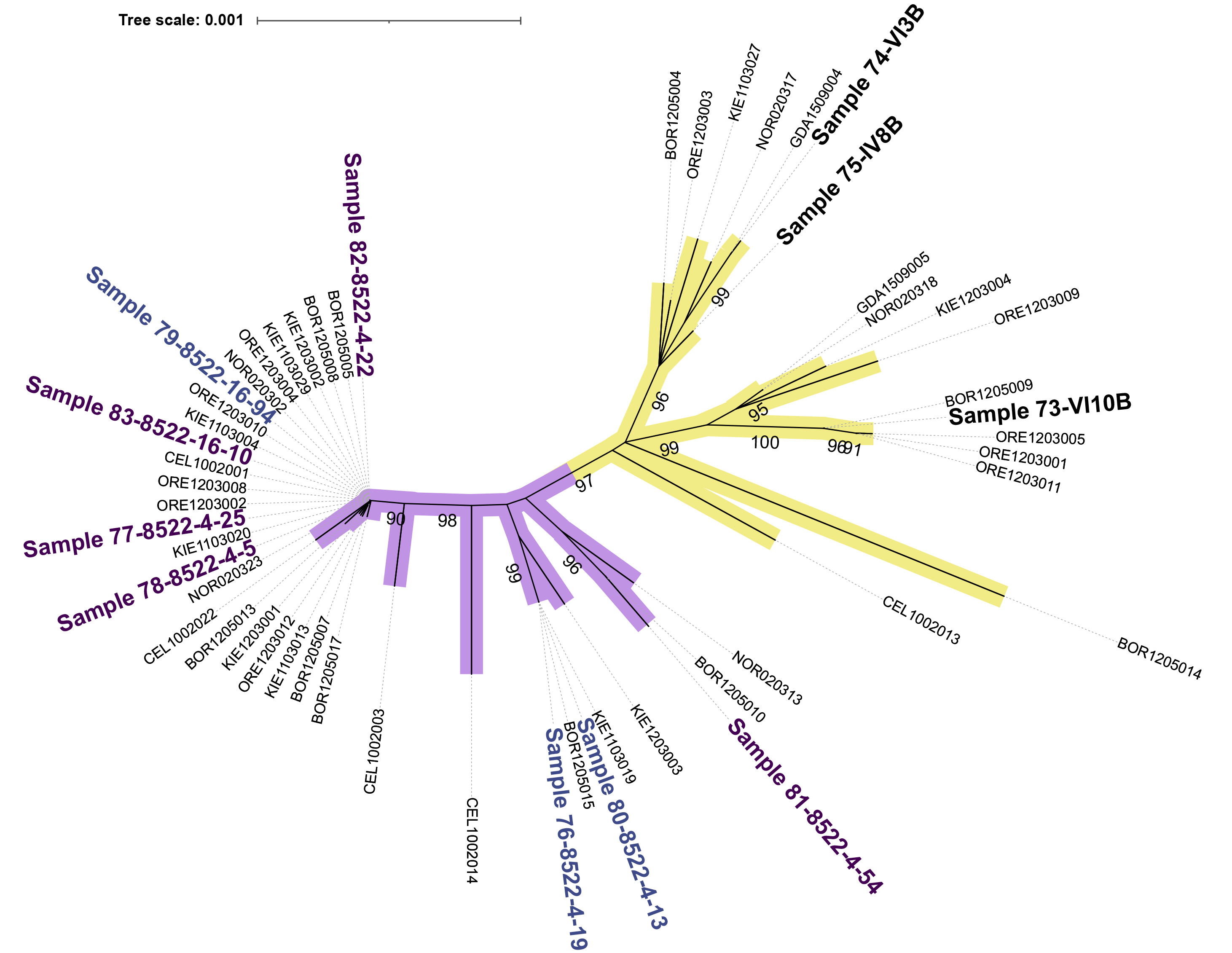


**Figure S7** Phylogenetic tree for whole mitochondrial genome, based on maximum likelihood analysis. Support values >90 are shown.

**2.5 MT genome analyses**


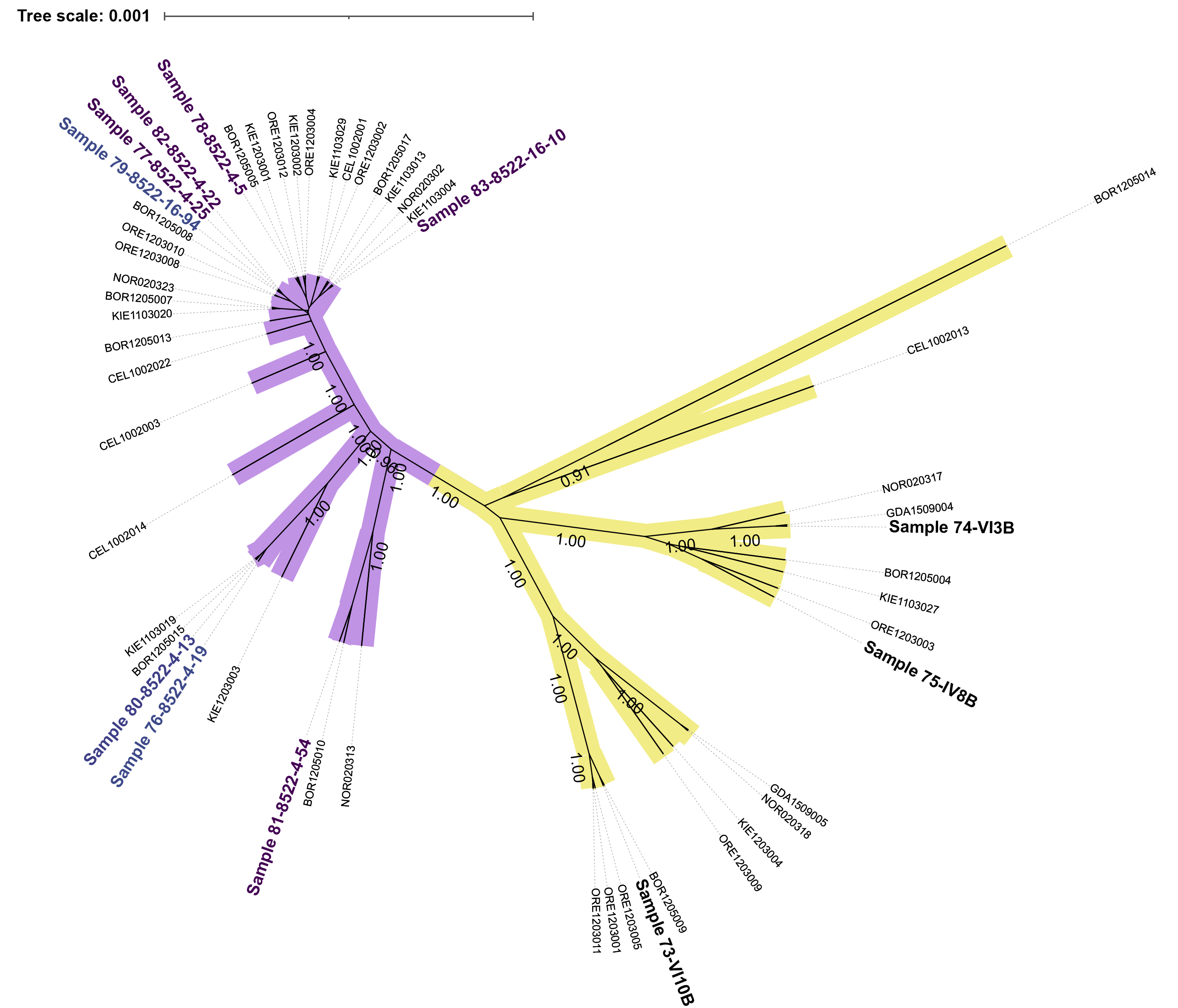


**Figure S8** Phylogenetic tree for whole mitochondrial genome, based on Bayesian analysis. Posterior probabilites >0.90 are shown.

#### **2.6 Inversion scoring**


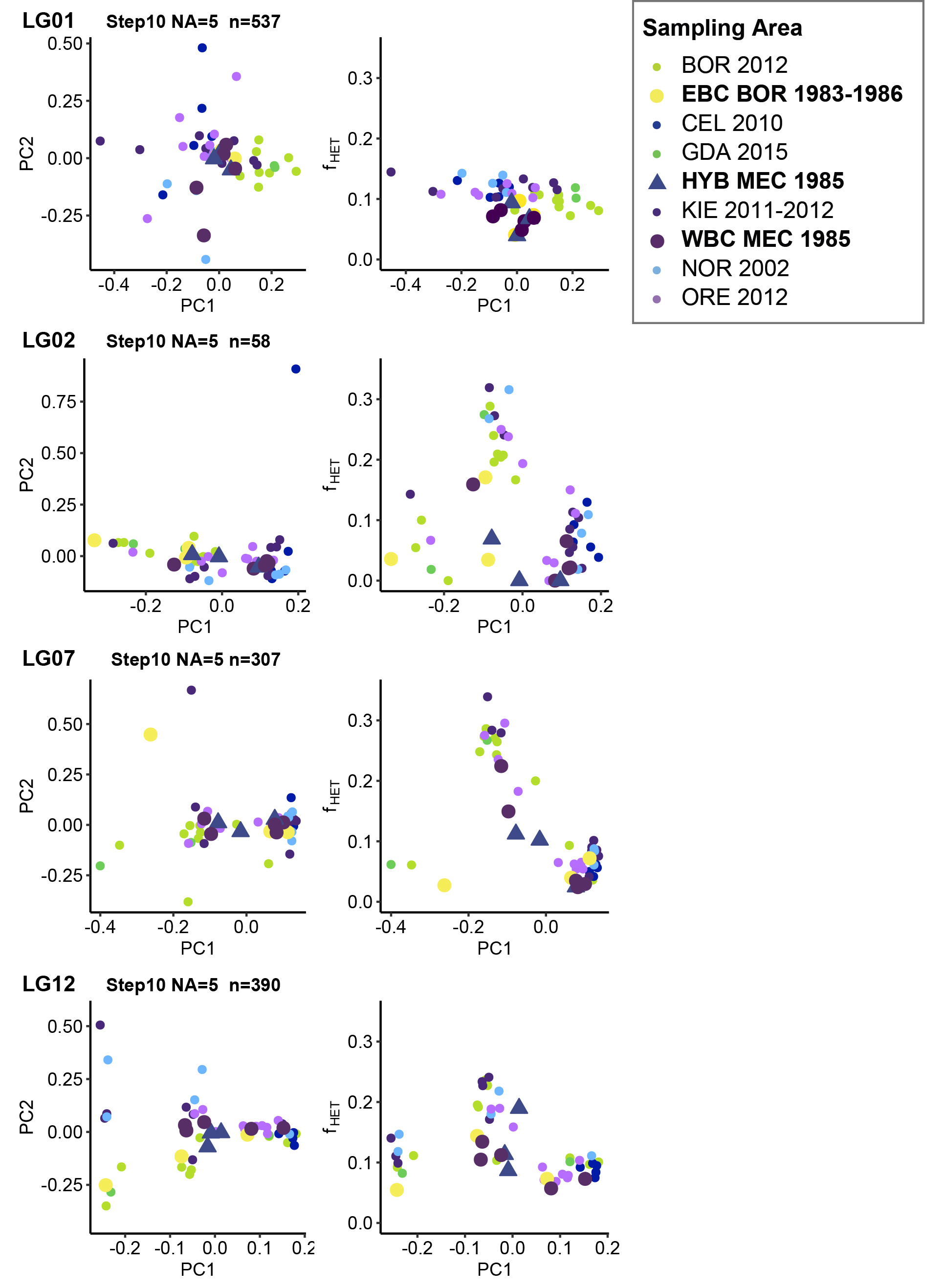


**Figure S9** PCA plots (PC1 vs PC2) and conformation of genotypes by plotting PC1 versus
f _HET_ (fraction heterozygosity). Plots made for sites within known inversions at LG01, LG02, LG07 and LG12. Filtering step and number of sites given at top of each PCA plot.

**2.6 Inversion scoring**


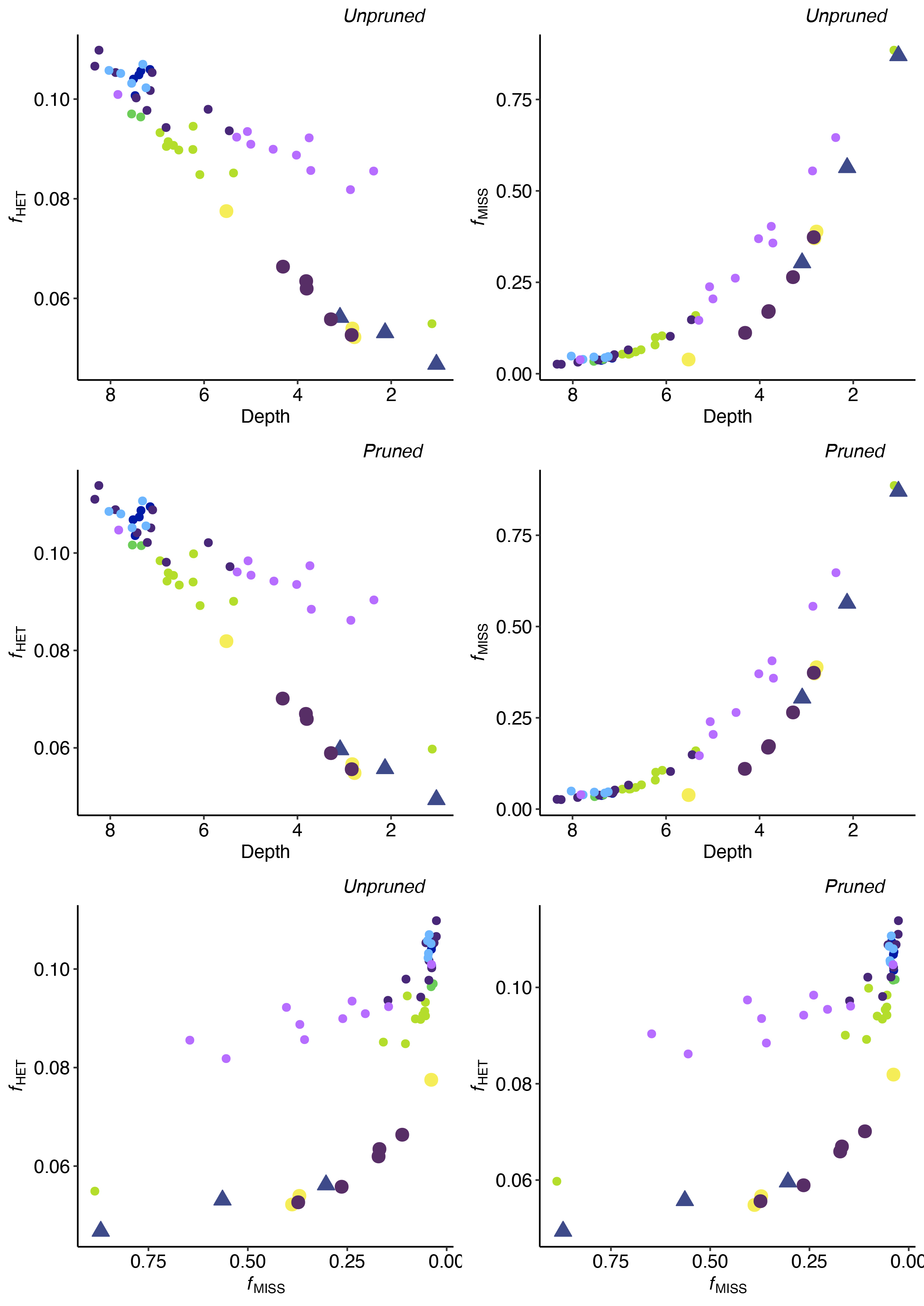

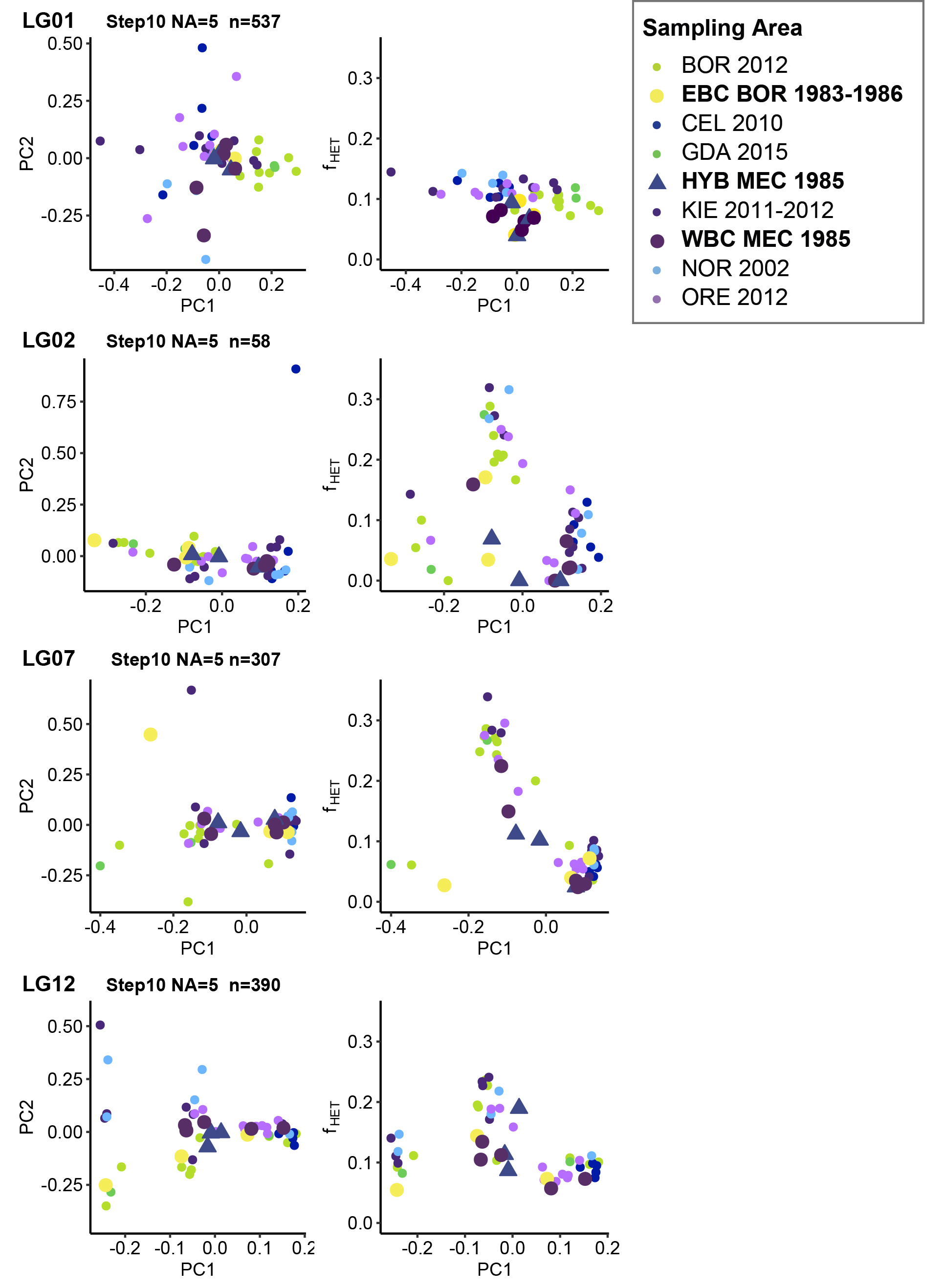


**Figure S10** Fraction of heterozygosity (f _HET_) and missing data (f _MISS_) in relation to depth and in relation to each other.

**Table S7** Comparison of genotypes at SNP sites within inversions present at linkage groups LG02, LG07 and LG12 in the SNP dataset versus those genotypes obtained from WGS dataset. A summary of matches and mismatches, as well as summarising which type of mismatch. Genotypes defined as in first 3 rows in the SNP set. Matching scoring marked with green. Error rate marked at bottom. Error rate calculated as number of matches divided by total. REF=reference genotype (as in gadMor2), NON_REF=non reference genotype and HET= heterozygous, NA=non applicable.

**2.6 Inversion scoring**

| ***Linkage group*** | | ***LG02*** | | ***LG07*** | | | ***LG12*** | |
| --- | --- | --- | --- | --- | --- | --- | --- | --- |
| ***Genotype*** | ***Position*** | ***18724285*** | ***20868512*** | ***14812281*** | ***16410308*** | ***20281433*** | ***11630885*** | ***12529238*** |
| **REF** | | A/A | T/T | T/T | A/A | C/C | G/G | T/T |
| **NON_REF** | | T/T | C/C | C/C | G/G | T/T | T/T | A/A |
| **HET** | | A/T | T/C | T/C | A/G | C/T | G/T | T/A |
| Number of NAs | | 1 | 7 | 3 | 0 | 0 | 3 | 2 |
| NON_REF called NON_REF | | 2 | 1 | 1 | 3 | 5 | 2 | 2 |
| NON_REF called as HET | | 2 | 0 | 1 | 1 | 1 | 1 | 1 |
| NON_REF called REF | | 0 | 0 | 1 | 0 | 0 | 0 | 0 |
| HET called HET | | 3 | 1 | 0 | 2 | 1 | 5 | 6 |
| HET called NON_REF | | 0 | 0 | 0 | 1 | 0 | 0 | 0 |
| HET called REF | | 2 | 1 | 2 | 0 | 1 | 0 | 0 |
| REF called REF | | 1 | 1 | 1 | 0 | 0 | 0 | 0 |
| REF called HET | | 0 | 0 | 0 | 1 | 1 | 0 | 0 |
| REF called NON_REF | | 0 | 0 | 1 | 0 | 1 | 0 | 0 |
| NA called REF | | 0 | 0 | 1 | 0 | 0 | 0 | 0 |
| NA called HET | | 0 | 0 | 0 | 1 | 1 | 0 | 0 |
| NA called NON_REF | | 0 | 0 | 0 | 2 | 0 | 0 | 0 |
| **Total** | | **11** | **11** | **11** | **11** | **11** | **11** | **11** |
| Mismatches | | 4 | 1 | 5 | 3 | 4 | 1 | 1 |
| Matches | | 6 | 3 | 2 | 5 | 6 | 7 | 8 |
| Missing | | 1 | 7 | 3 | 0 | 0 | 3 | 2 |
| **Error-rate** | | **0.36** | **0.09** | **0.45** | **0.27** | **0.36** | **0.09** | **0.09** |

**2.6 Inversion scoring**

**Table S8** Comparison of genotypes at SNP site with 9% error rate in historical SNP data (right column) with scoring in purely modern samples for LG02 SNP at same SNP position present in the WGS data.

|  | ***Dataset*** | ***Modern*** | ***Historical*** |
| --- | --- | --- | --- |
| ***Genotype*** | ***Site*** | ***LG02_20868512*** | ***LG02_20868512*** |
| **REF** | | 0/0 or T/T | T/T |
| **NON_REF** | | 1/1 or C/C | C/C |
| **HET** | | 0/1 or T/C | T/C |
| Number of NAs | | 3 | 7 |
| NON_REF called NON_REF | | 19 | 1 |
| NON_REF called as HET | | 0 | 0 |
| NON_REF called REF | | 0 | 0 |
| HET called HET | | 12 | 1 |
| HET called NON_REF | | 0 | 0 |
| HET called REF | | 2 | 1 |
| REF called REF | | 6 | 1 |
| REF called HET | | 0 | 0 |
| REF called NON_REF | | 0 | 0 |
| NA called REF | | 0 | 0 |
| NA called HET | | 0 | 0 |
| NA called NON_REF | | 0 | 0 |
| **Total** | | **42** | **11** |
| Mismatches | | 2 | 1 |
| Matches | | 37 | 3 |
| Missing | | 3 | 7 |
| **Error-rate** | | **0.05** | **0.09** |
|  |  |  |  |
|  |  | ***Historical N=436*** |  |
|  |  | ***LG02_20868512*** |  |
|  | **NA** | **68** |  |
|  | **Genotyped** | **368** |  |

**Table S9** Comparison of genotypes at two SNP sites with 9% error rate in historical SNP data (right columns) with scoring in purely modern samples for LG12 SNPs at same SNP positions present in the WGS data.

|  | ***Historical N=436*** | |
| --- | --- | --- |
|  | ***LG12_11630885***  ***_I12*** | ***LG12_12529238***  ***_I12*** |
| **NA** | **53** | 90 |
| **Genotyped** | **383** | 346 |

|  |  | ***Modern*** | | ***Historical*** | |  |
| --- | --- | --- | --- | --- | --- | --- |
| ***Genotype*** | ***Site*** | ***LG12_11630885*** | ***LG12_12529238*** | ***LG12_***  ***11630885_I12*** | ***LG12_***  ***12529238_I12*** |  |
| **REF** | | 0/0 or G/G | 0/0 or T/T | G/G | T/T |  |
| **NON_REF** | | 1/1 or T/T | 1/1 or A/A | T/T | A/A |  |
| **HET** | | 0/1 or G/T | 0/1 or T/A | G/T | T/A |  |
| Number of NAs | | 1 | 2 | 3 | 2 |  |
| NON_REF called NON_REF | | 19 | 19 | 2 | 2 |  |
| NON_REF called as HET | | 1 | 1 | 1 | 1 |  |
| NON_REF called REF | | 0 | 0 | 0 | 0 |  |
| HET called HET | | 12 | 11 | 5 | 6 |  |
| HET called NON_REF | | 1 | 0 | 0 | 0 |  |
| HET called REF | | 0 | 1 | 0 | 0 |  |
| REF called REF | | 8 | 8 | 0 | 0 |  |
| REF called HET | | 0 | 0 | 0 | 0 |  |
| REF called NON_REF | | 0 | 0 | 0 | 0 |  |
| NA called REF | | 0 | 0 | 0 | 0 |  |
| NA called HET | | 0 | 0 | 0 | 0 |  |
|  | NA called NON_ REF | 0 | 0 | 0 | 0 |  |
|  | **Total** | **42** | **42** | **11** | **11** |  |
|  | Mismatches | 2 | 2 | 1 | 1 |  |
|  | Matches | 39 | 38 | 7 | 8 |  |
|  | Missing | 1 | 2 | 2 | 2 |  |
|  | **Error-rate** | **0.05** | **0.05** | **0.09** | **0.09** |  |

# **3. Results**

#### **3.1 Population structure and hybrid assessment**

**Table S10** Tracy Windom statistics for 19 SNP dataset.

| ***Component*** | ***Eigenvalue*** | ***difference*** | ***TW-stat*** | ***p-value*** | ***effect.n*** |
| --- | --- | --- | --- | --- | --- |
| **1** | **39.152356** | **NA** | **2.182** | **0.00762443** | **8.982** |
| 2 | 7.338790 | -31.813566 | -2.017 | 0.730539 | 58.167 |
| 3 | 7.304283 | -0.034508 | -0.807 | 0.359124 | 57.609 |
| 4 | 6.120227 | -1.184055 | -1.613 | 0.609428 | 61.590 |
| 5 | 5.765593 | -0.354634 | -1.037 | 0.427396 | 62.445 |
| 6 | 4.671237 | -1.094356 | -2.361 | 0.818384 | 66.243 |
| 7 | 4.190343 | -0.480893 | -2.714 | 0.888791 | 63.810 |
| 8 | 4.073530 | -0.116813 | -2.109 | 0.755587 | 59.546 |
| 9 | 3.752035 | -0.321496 | -1.948 | 0.710915 | 57.221 |
| 10 | 3.457288 | -0.294747 | -1.679 | 0.630292 | 55.162 |
| 11 | 2.997161 | -0.460127 | NA | NA | NA |
| 12 | 2.918535 | -0.078626 | NA | NA | NA |
| 13 | 2.176099 | -0.742436 | NA | NA | NA |
| 14 | 1.905457 | -0.270642 | NA | NA | NA |
| 15 | 1.890940 | -0.014517 | NA | NA | NA |
| 16 | 1.602450 | -0.288490 | NA | NA | NA |
| 17 | 1.378144 | -0.224306 | NA | NA | NA |
| 18 | 0.976193 | -0.401951 | NA | NA | NA |
| 19 | 0.329339 | -0.646854 | NA | NA | NA |

**3.1 Population structure and hybrid assessment**


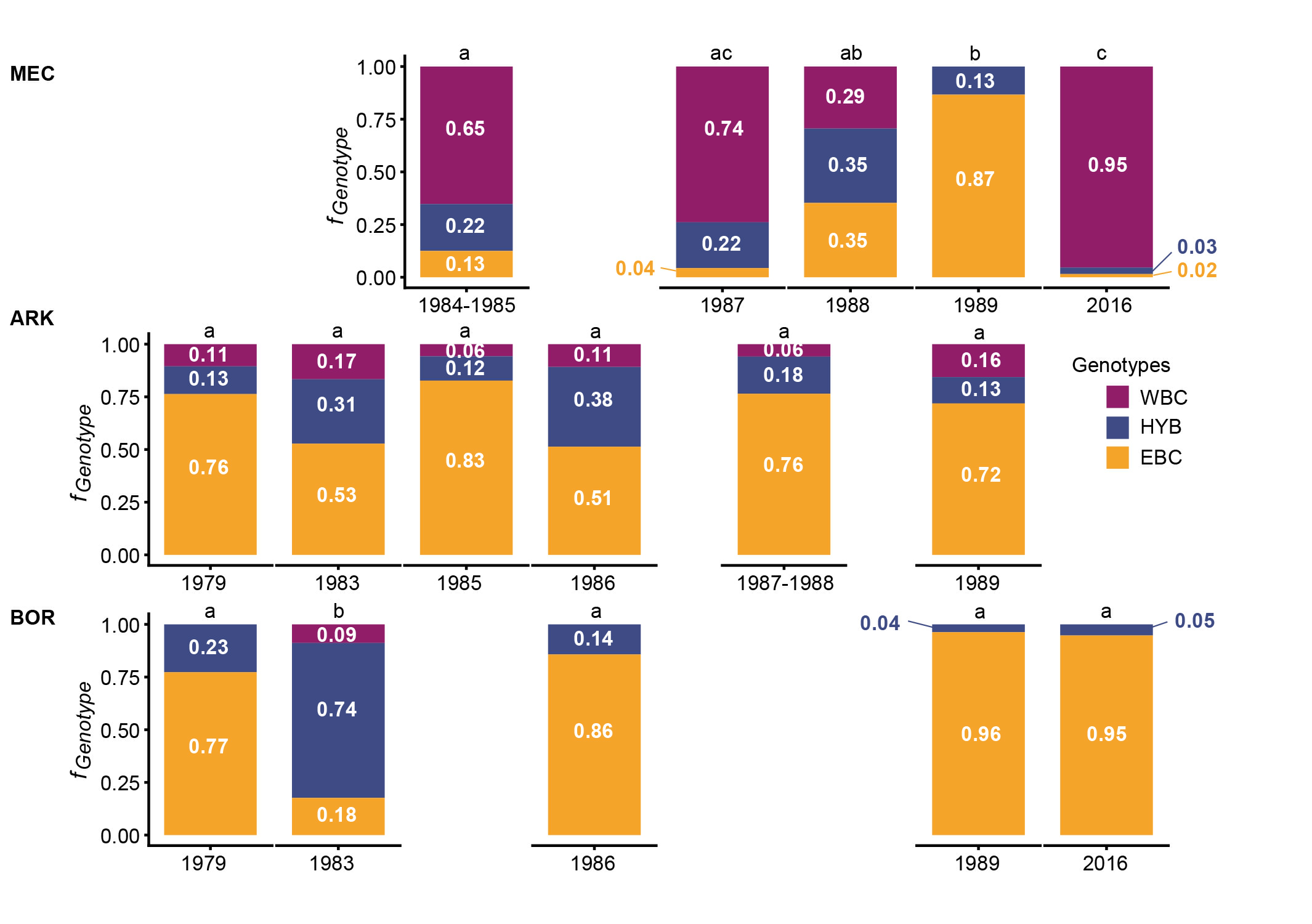


**Figure S11** Proportion of STRUCTURE genetically assigned WBC, HYB and EBC in three different areas. Letters a,b,c showing significantly different groups in pairwise comparisons within each area after Bonferroni correction at p<0.05.

**3.1 Population structure and hybrid assessment**

**Table S11** Fisher´s exact test for difference between proportion of Baltic cod types over time in different areas. Significant p-values (p<0.05) in red.

| ***Area*** | ***p*** |
| --- | --- |
| MEC | 2.20e-16 |
| ARK | 3.35e-02 |
| BOR | 3.30e-12 |

**Table S12** Fisher´s exact test for pairwise difference between proportion of Baltic cod types over time in MEC. Significant p-values (p<0.05 after correction for multiple testing) in red. Low p-values but not significant due to multiple testing in brown.

| ***MEC Comparison*** | | ***p*** |
| --- | --- | --- |
| 1 | 1984-85 vs 1987 | 0.65 |
| 2 | 1984-85 vs 1988 | 1.29E-02 |
| 3 | 1984-85 vs 1989 | 5.55E-09 |
| 4 | 1984-85 vs 2016 | 2.70E-05 |
| 5 | 1987 vs 1988 | 8.14E-03 |
| 6 | 1987 vs 1989 | 2.93E-08 |
| 7 | 1987 vs 2016 | 8.67E-03 |
| 8 | 1988 vs 1989 | 8.44E-03 |
| 9 | 1988 vs 2016 | 2.76E-08 |
| 10 | 1989 vs 2016 | 2.26E-14 |

**Table S13** Fisher´s exact test for pairwise difference between proportion of Baltic cod types over time in ARK. Significant p-values (p<0.05 after correction for multiple testing) in red. Low p-values but not significant due to multiple testing in brown.

| ***ARK Comparison*** | | ***p*** |
| --- | --- | --- |
| 1 | 1979 vs 1983 | 0.10 |
| 2 | 1979 vs 1985 | 0.68 |
| 3 | 1979 vs 1986 | 0.04 |
| 4 | 1979 vs 1987-88 | 0,89 |
| 5 | 1979 vs 1989 | 0.92 |
| 6 | 1983 vs 1985 | 0.01 |
| 7 | 1983 vs 1986 | 0.73 |
| 8 | 1983 vs 1987-88 | 0.33 |
| 9 | 1983 vs 1989 | 0.19 |
| 10 | 1985 vs 1986 | 4.54E-03 |
| 11 | 1985 vs 1987-88 | 0.86 |
| 12 | 1985 vs 1989 | 0.32 |
| 13 | 1986_vs 1987-88 | 0.20 |
| 14 | 1986 vs 1989 | 0.06 |
| 15 | 1987-88 vs 1989 | 0.71 |

**3.1 Population structure and hybrid assessment**

**Table S14** Fisher´s exact test for pairwise difference between proportion of Baltic cod types over time in BOR. Significant p-values (p<0.05 after correction for multiple testing) in red.

| ***BOR* *Comparison*** | | ***p*** |
| --- | --- | --- |
| 1 | 1979 vs 1983 | 2.60E-05 |
| 2 | 1979 vs 1986 | 0.68 |
| 3 | 1979 vs 1989 | 0.21 |
| 4 | 1979 vs 2016 | 0.09 |
| 5 | 1983 vs 1986 | 2.51E-05 |
| 6 | 1983 vs 1989 | 2.05E-07 |
| 7 | 1983 vs 2016 | 9.56E-12 |
| 8 | 1986 vs 1989 | 0.58 |
| 9 | 1986 vs 2016 | 0.29 |
| 10 | 1989 vs 2016 | 1.00 |

**3.1 Population structure and hybrid assessment**

**Table S15** Tracy Windom statistics for low coverage WGS dataset.

| ***Component*** | ***Eigenvalue*** | ***difference*** | ***TW-stat*** | ***p-value*** | ***effect.n*** |
| --- | --- | --- | --- | --- | --- |
| **1** | **1.905864** | **NA** | **2.146** | **0.00812358** | **419.519** |
| 2 | 1.424173 | -0.481692 | -9.332 | 1 | 445.273 |
| 3 | 1.405561 | -0.018612 | -9.385 | 1 | 435.443 |
| 4 | 1.396367 | -0.009194 | -9.179 | 1 | 425.305 |
| 5 | 1.392049 | -0.004318 | -8.831 | 1 | 415.332 |
| 6 | 1.363197 | -0.028852 | -9.138 | 1 | 405.800 |
| 7 | 1.355818 | -0.007379 | -8.876 | 1 | 395.462 |
| 8 | 1.348872 | -0.006946 | -8.591 | 1 | 385.398 |
| 9 | 1.338068 | -0.010804 | -8.395 | 1 | 375.657 |
| 10 | 1.331016 | -0.007052 | -8.091 | 1 | 366.065 |
| 11 | 1.321014 | -0.010003 | -7.848 | 1 | 356.848 |
| 12 | 1.307669 | -0.013344 | -7.681 | 1 | 347.883 |
| 13 | 1.294007 | -0.013662 | -7.513 | 1 | 339.013 |
| 14 | 1.285556 | -0.008451 | -7.201 | 1 | 330.239 |
| 15 | 1.283289 | -0.002268 | -6.708 | 1 | 321.892 |
| 16 | 1.266650 | -0.016638 | -6.552 | 1 | 314.429 |
| 17 | 1.226191 | -0.040459 | -7.003 | 1 | 307.047 |
| 18 | 1.223482 | -0.002710 | -6.519 | 1 | 298.232 |
| 19 | 1.203073 | -0.020409 | -6.453 | 1 | 290.299 |
| 20 | 1.166409 | -0.036664 | -6.799 | 1 | 282.200 |
| 21 | 1.117052 | -0.049357 | -7.494 | 1 | 272.885 |
| 22 | 1.102296 | -0.014755 | -7.365 | 1 | 261.652 |
| 23 | 1.057521 | -0.044775 | -7.964 | 1 | 250.433 |
| 24 | 1.053918 | -0.003604 | -7.592 | 1 | 237.774 |
| 25 | 1.036810 | -0.017108 | -7.520 | 1 | 225.641 |
| 26 | 1.033028 | -0.003781 | -7.134 | 1 | 213.455 |
| 27 | 1.003986 | -0.029042 | -7.299 | 1 | 201.828 |
| 28 | 0.991497 | -0.012490 | -7.099 | 1 | 189.699 |
| 29 | 0.988244 | -0.003253 | -6.688 | 1 | 177.786 |
| 30 | 0.978822 | -0.009421 | -6.385 | 1 | 166.482 |
| 31 | 0.971505 | -0.007318 | -6.020 | 0.999998 | 155.582 |
| 32 | 0.962292 | -0.009212 | -5.667 | 0.999985 | 145.211 |
| 33 | 0.931417 | -0.030875 | -5.707 | 0.999988 | 135.349 |
| 34 | 0.927828 | -0.003589 | -5.230 | 0.999891 | 125.151 |
| 35 | 0.888796 | -0.039032 | -5.364 | 0.999939 | 115.739 |
| 36 | 0.865774 | -0.023022 | -5.217 | 0.999885 | 105.786 |
| 37 | 0.853598 | -0.012177 | -4.866 | 0.999535 | 95.944 |
| 38 | 0.845914 | -0.007683 | -4.409 | 0.997735 | 86.625 |
| 39 | 0.830777 | -0.015138 | -4.023 | 0.992895 | 78.061 |
| 40 | 0.813169 | -0.017608 | -3.626 | 0.980546 | 70.102 |
| 41 | 0.807609 | -0.005560 | -2.986 | 0.928654 | 62.795 |
| 42 | 0.793648 | -0.013961 | -2.332 | 0.81173 | 56.931 |
| 43 | 0.660111 | -0.133537 | -3.305 | 0.960893 | 52.969 |
| 44 | 0.622518 | -0.037592 | NA | NA | NA |
| 45 | 0.579449 | -0.043069 | NA | NA | NA |
| 46 | 0.552093 | -0.027356 | NA | NA | NA |
| 47 | 0.539126 | -0.012968 | NA | NA | NA |
| 48 | 0.500902 | -0.038223 | NA | NA | NA |
| 49 | 0.452915 | -0.047988 | NA | NA | NA |
| 50 | 0.213770 | -0.239144 | NA | NA | NA |
| 51 | 0.115058 | -0.098712 | NA | NA | NA |
| 52 | 0.070230 | -0.044828 | NA | NA | NA |

**3.1 Population structure and hybrid assessment**

**Table S16** ANOVA statistics (EIG v.7.2.1) stating groups of comparison (Sampling area), p-value and significance level. Significance level given as p-values less than 0.000001+++, between 0.000001 and 0.001***, between 0.001 and 0.01** and as between 0.01 and 0.05*. Symbols as in PCA plots.

| ***ANOVA statistics*** | | | |
| --- | --- | --- | --- |
| **Sampling area 1** | **Sampling area 2** | **p-value** | **Significance level** |
| ⚫ BOR 2012 | ⚫ CEL 2010 | 1.61438e-08 | +++ |
| ⚫ BOR 2012 | ⚫ GDA 2015 | 0.729071 |  |
| ⚫ BOR 2012 | ⚫ KIE 2011-2012 | 5.82475e-06 | *** |
| ⚫ BOR 2012 | ⚫ NOR 2002 | 2.37715e-08 | +++ |
| ⚫ BOR 2012 | ⚫ ORE 2012 | 1.5126e-08 | +++ |
| ⚫ BOR 2012 | ⚫ EBC BOR 1983-86 | 0.0996427 |  |
| ⚫ BOR 2012 | **▲** HYB KIE 1985 | 0.00133974 | ** |
| ⚫ BOR 2012 | ⚫ WBC KIE 1985 | 8.40691e-06 | *** |
| ⚫ CEL 2010 | ⚫ GDA 2015 | 6.50765e-06 | *** |
| ⚫ CEL 2010 | ⚫ KIE 2011-2012 | 0.00111915 | ** |
| ⚫ CEL 2010 | ⚫ NOR 2002 | 0.497712 |  |
| ⚫ CEL 2010 | ⚫ ORE 2012 | 1.17698e-10 | +++ |
| ⚫ CEL 2010 | ⚫ EBC BOR 1983-86 | 2.74629e-05 | *** |
| ⚫ CEL 2010 | **▲** HYB KIE 1985 | 1.16177e-05 | *** |
| ⚫ CEL 2010 | ⚫ WBC KIE 1985 | 7.45784e-07 | +++ |
| ⚫ GDA 2015 | ⚫ KIE 2011-2012 | 0.00406237 | ** |
| ⚫ GDA 2015 | ⚫ NOR 2002 | 8.51479e-06 | *** |
| ⚫ GDA 2015 | ⚫ ORE 2012 | 1.60147e-09 | +++ |
| ⚫ GDA 2015 | ⚫ EBC BOR 1983-86 | 0.108121 |  |
| ⚫ GDA 2015 | **▲** HYB KIE 1985 | 0.00127813 | ** |
| ⚫ GDA 2015 | ⚫ WBC KIE 1985 | 5.03822e-06 | *** |
| ⚫ KIE 2011-2012 | ⚫ NOR 2002 | 0.00193853 | ** |
| ⚫ KIE 2011-2012 | ⚫ ORE 2012 | 0.711646 |  |
| ⚫ KIE 2011-2012 | ⚫ EBC BOR 1983-86 | 0.0225648 | * |
| ⚫ KIE 2011-2012 | **▲** HYB KIE 1985 | 0.372669 |  |
| ⚫ KIE 2011-2012 | ⚫ WBC KIE 1985 | 0.963806 |  |
| ⚫ NOR 2002 | ⚫ ORE 2012 | 3.46614e-10 | +++ |
| ⚫ NOR 2002 | ⚫ EBC BOR 1983-86 | 3.61081e-05 | *** |
| ⚫ NOR 2002 | **▲** HYB KIE 1985 | 1.86154e-05 | *** |
| ⚫ NOR 2002 | ⚫ WBC KIE 1985 | 1.56536e-06 | *** |
| ⚫ ORE 2012 | ⚫ EBC BOR 1983-86 | 8.30991e-06 | *** |
| ⚫ ORE 2012 | **▲** HYB KIE 1985 | 0.00123985 | ** |
| ⚫ ORE 2012 | ⚫ WBC KIE 1985 | 0.225824 |  |
| ⚫ EBC BOR 1983-85 | **▲** HYB KIE 1985 | 0.0422896 | * |
| ⚫ EBC BOR 1983-85 | ⚫ WBC KIE 1985 | 0.000971752 | *** |
| **▲** HYB KIE 1985 | ⚫ WBC KIE 1985 | 0.00186617 | ** |

**3.1 Population structure and hybrid assessment**

**Table S17** Simplified summary of ANOVA statistics, with rounded off p-values, to two decimals. 0.00 is shown as 0.

| ***ANOVA statsictics (p-values)*** | | | | | | | | |
| --- | --- | --- | --- | --- | --- | --- | --- | --- |
| ⚫ **NOR** | ⚫ **ORE** | ⚫ **KIE** | ⚫ **BOR** | ⚫ **GDA** | ⚫ **WBC** | **▲ HYB** | ⚫ **EBC** | ***Group*** |
| 0.50 | 0 | 0 | 0 | 0 | 0 | 0 | 0 | ⚫ **CEL** |
|  | 0 | 0 | 0 | 0 | 0 | 0 | 0 | ⚫ **NOR** |
|  |  | 0.71 | 0 | 0 | 0.23 | 0 | 0 | ⚫ **ORE** |
|  |  |  | 0 | 0 | 0.96 | 0.37 | 0.02 | ⚫ **KIE** |
|  |  |  |  | 0.73 | 0 | 0 | 0.10 | ⚫ **BOR** |
|  |  |  |  |  | 0 | 0 | 0.11 | ⚫ **GDA** |
|  |  |  |  |  |  | 0 | 0 | ⚫ **WBC** |
|  |  |  |  |  |  |  | 0.04 | **▲ HYB** |

**3.1 Population structure and hybrid assessment**

**Table S18** Chi-square statistics for WGS dataset.

| ***pop1*** | ***pop2*** | *χ* ***2*** |  | ***N (pop1)*** | ***N (pop2)*** |
| --- | --- | --- | --- | --- | --- |
|  |  |  | ***p-value*** |  |  |
| ⚫ BOR 2012 | ⚫ CEL 2010 | 44.372 | 1.94642e-08 | 10 | 5 |
| ⚫ BOR 2012 | ⚫ GDA 2015 | 2.775 | 0.734605 | 10 | 2 |
| **⚫ BOR 2012** | **⚫ KIE 2011-2012** | **44.785** | **1.60482e-08** | **10** | **10** |
| ⚫ BOR 2012 | ⚫ NOR 2002 | 45.765 | 1.0141e-08 | 10 | 5 |
| ⚫ BOR 2012 | ⚫ ORE 2012 | 65.367 | 9.40502e-13 | 10 | 10 |
| **⚫ BOR 2012** | **⚫ EBC BOR 1983-86** | **8.128** | **0.149307** | **10** | **3** |
| **⚫ BOR 2012** | **▲ HYB KIE 1985** | **22.784** | **0.000371141** | **10** | **3** |
| **⚫ BOR 2012** | **⚫ WBC KIE 1985** | **40.205** | **1.35764e-07** | **10** | **5** |
| ⚫ CEL 2010 | ⚫ GDA 2015 | 31.947 | 6.08706e-06 | 5 | 2 |
| ⚫ CEL 2010 | ⚫ KIE 2011-2012 | 24.499 | 0.000174087 | 5 | 10 |
| ⚫ CEL 2010 | ⚫ NOR 2002 | 6.691 | 0.244642 | 5 | 5 |
| ⚫ CEL 2010 | ⚫ ORE 2012 | 57.922 | 3.26439e-11 | 5 | 10 |
| ⚫ CEL 2010 | ⚫ EBC BOR 1983-86 | 27.126 | 5.39124e-05 | 5 | 3 |
| ⚫ CEL 2010 | **▲** HYB KIE 1985 | 29.673 | 1.71049e-05 | 5 | 3 |
| ⚫ CEL 2010 | ⚫ WBC KIE 1985 | 36.163 | 8.81282e-07 | 5 | 5 |
| ⚫ GDA 2015 | ⚫ KIE 2011-2012 | 14.042 | 0.0153453 | 2 | 10 |
| ⚫ GDA 2015 | ⚫ NOR 2002 | 31.427 | 7.71461e-06 | 2 | 5 |
| ⚫ GDA 2015 | ⚫ ORE 2012 | 54.965 | 1.32749e-10 | 2 | 10 |
| ⚫ GDA 2015 | ⚫ EBC BOR 1983-86 | 5.704 | 0.336109 | 2 | 3 |
| ⚫ GDA 2015 | **▲** HYB KIE 1985 | 28.979 | 2.34038e-05 | 2 | 3 |
| ⚫ GDA 2015 | ⚫ WBC KIE 1985 | 41.421 | 7.71183e-08 | 2 | 5 |
| ⚫ KIE 2011-12 | ⚫ NOR 2002 | 17.033 | 0.00443803 | 10 | 5 |
| ⚫ KIE 2011-12 | ⚫ ORE 2012 | 9.345 | 0.0960849 | 10 | 10 |
| **⚫ KIE 2011-12** | **⚫ EBC BOR 1983-86** | **11.905** | **0.0361154** | **10** | **3** |
| **⚫ KIE 2011-12** | **▲ HYB KIE 1985** | **5.188** | **0.393422** | **10** | **3** |
| **⚫ KIE 2011-12** | **⚫ WBC KIE 1985** | **6.692** | **0.244593** | **10** | **5** |
| ⚫ NOR 2002 | ⚫ ORE 2012 | 56.395 | 6.73987e-11 | 5 | 10 |
| ⚫ NOR 2002 | ⚫ EBC BOR 1983-86 | 26.892 | 5.98645e-05 | 5 | 3 |
| ⚫ NOR 2002 | **▲** HYB KIE 1985 | 28.701 | 2.65393e-05 | 5 | 3 |
| ⚫ NOR 2002 | ⚫ WBC KIE 1985 | 34.656 | 1.76186e-06 | 5 | 5 |
| ⚫ ORE 2012 | ⚫ EBC BOR 1983-86 | 37.372 | 5.04484e-07 | 10 | 3 |
| ⚫ ORE 2012 | **▲** HYB KIE 1985 | 19.094 | 0.00184632 | 10 | 3 |
| ⚫ ORE 2012 | ⚫ WBC KIE 1985 | 11.181 | 0.0479153 | 10 | 5 |
| **⚫ EBC BOR 1983-86** | **▲ HYB KIE 1985** | **13.989** | **0.0156793** | **3** | **3** |
| **⚫ EBC BOR 1983-86** | **⚫ WBC KIE 1985** | **23.238** | **0.00030402** | **3** | **5** |
| **▲ HYB KIE 1985** | **⚫ WBC KIE 1985** | **15.480** | **0.00849802** | **3** | **5** |

**3.1 Population structure and hybrid assessment**

**Table S19** List of SNPs driving first principal component, sorted by increasing SNP-weight for the 19 SNP dataset.

| **Marker ID** | **LG** | **Position** | **SNP weight** |
| --- | --- | --- | --- |
| LG01_10417249_SEL | 1 | 10417249 | 0.934 |
| LG21_18500787_SEL | 21 | 18500787 | 0.965 |
| LG04_23059256_SEL_60 | 4 | 23059256 | 0.982 |
| LG02_14506653_SEL | 2 | 14506653 | 1.173 |
| LG06_00784232_SEL | 6 | 784232 | 1.184 |
| LG21_04164158_SEL | 21 | 4164158 | 1.245 |
| LG09_08678526_SEL | 9 | 8678526 | 1.335 |
| LG02_01358822_SEL | 2 | 1358822 | 1.358 |
| **LG12_07553923_SEL** | **12** | **7553923** | **1.408** |
| **LG11_20277669_SEL** | **11** | **20277669** | **1.538** |

**3.1 Population structure and hybrid assessment**

**Table S20** List of sites driving PCA in WGS dataset on the first principal component, position, search range in gadMor2 and annotation and type of match.

| *Site* | *Search Range (2kb)* | *Match* | *Type of match* |
| --- | --- | --- | --- |
| LG01:27861337 | 27860337 to 27862337 | Protein of unknown function LG01:27845265..27868932 (- strand) | Intron |
| LG03:10328516 | 10327516 to 10329516 | Protein of unknown function LG03:10327279..10329819 (- strand), Similar to Fam20a: Pseudokinase FAM20A (Mus musculus) LG03:10327265..10328118 (+ strand) | Intron |
| LG03:7117061 | 7116061 to 7118061 | Protein of unknown function LG03:7088952..7138297 (- strand) | Intron |
| LG08:17583281 | 17582281 to 17584281 | Similar to rx2: Retinal homeobox protein Rx2 (Oryzias latipes) LG08:17579097..17589036 (- strand) | Intron |
| LG11:20315510 | 20314510 to 20316510 | Similar to COLEC10: Collectin-10 (Gallus gallus) LG11:20313049..20314978 (+ strand), Similar to MAL2: Protein MAL2 (Homo sapiens) LG11:20316411..20324308 (+ strand) | Between two genes |
| LG11:10159388 | 10158388 to 10160388 | Similar to SEMA7A: Semaphorin-7A (Homo sapiens) LG11:10139780..10165455 (- strand) | Intron |
| LG18:20495510 | 20494510 to 20496510 | Protein of unknown function LG18:20495014..20496915 (+ strand) | Intron |
| LG18:4132249 | 4131249 to 4133249 | Similar to Adam11: Disintegrin and metalloproteinase domain-containing protein 11 (Mus musculus) LG18:4118161..4149905 (- strand) | Intron |
| LG23:5876035 | 5875035 to 5877035 | Similar to TFRC: Transferrin receptor protein 1 (Pongo abelii) LG23:5876501..5895590 (+ strand) | Outside gene |
| LG23:5195866 | 5194866 to 5196866 | Similar to ACOT9: Acyl-coenzyme A thioesterase 9, mitochondrial (Bos taurus) LG23:5194386..5204141 (+ strand) | Intron |

**3.1 Population structure and hybrid assessment**

**Table S21** Summary of statistics of test of difference in heterozygosity between cod types (WBC, HYB and EBC) and dataset (historical and modern).

| ***ANOVA statistics (Two-way)*** | | | | | |
| --- | --- | --- | --- | --- | --- |
|  | **df** | **Sum Sq** | **Mean Sq** | **F value** | **Pr(>F)** |
| **TYPE** | **2** | **4.340** | **2.1702** | **113.622** | **<2e-16***** |
| **DATASET** | 1 | 0.018 | 0.0178 | 0.929 | 0.335 |
| **TYPE*DATASET** | 2 | 0.045 | 0.0225 | 1.179 | 0.308 |
| **Residuals** | 533 | 10.180 | 0.0191 |  |  |


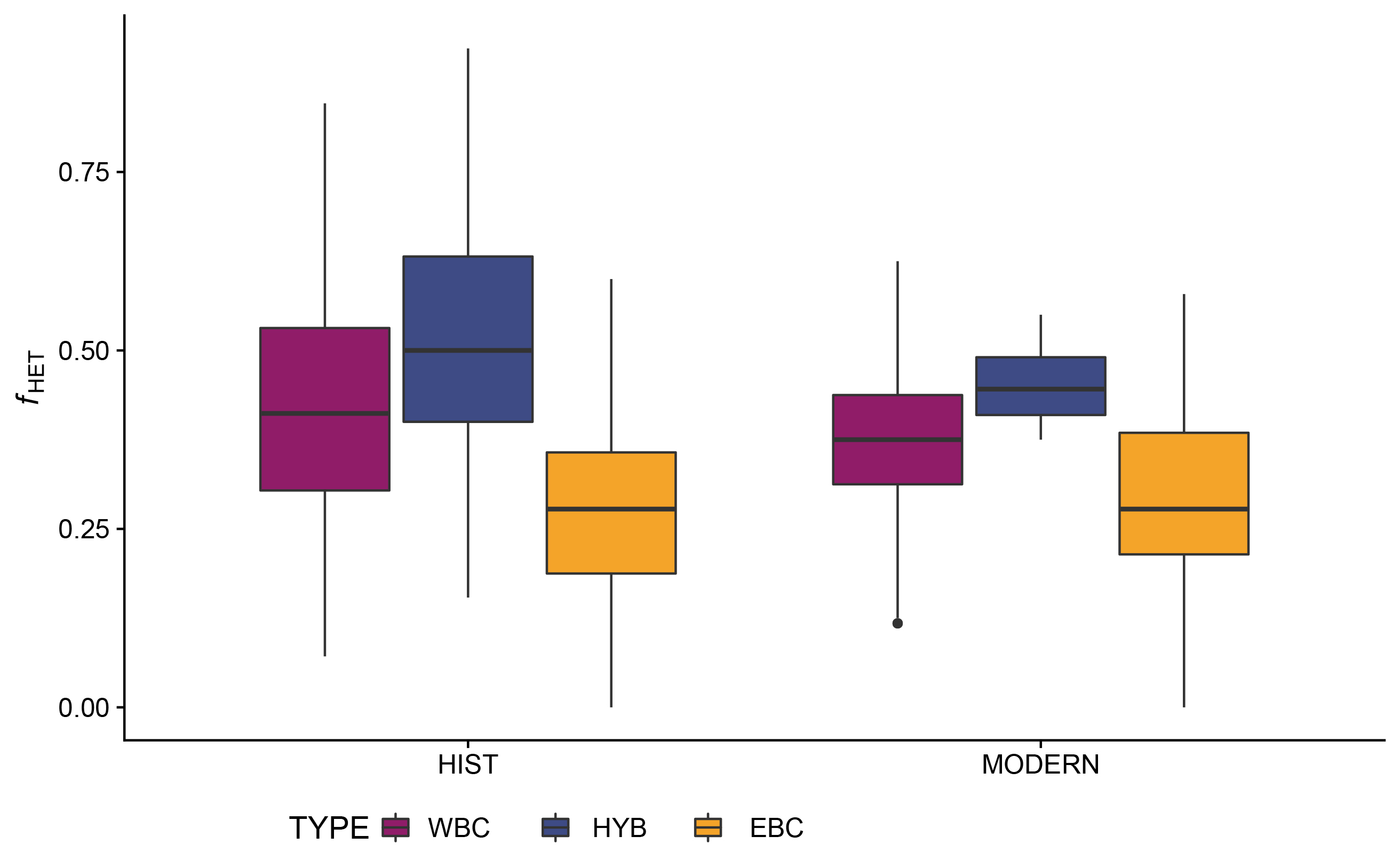


**Figure S12** Boxplot for Two-way ANOVA for 20 SNP dataset.

**3.1 Population structure and hybrid assessment**


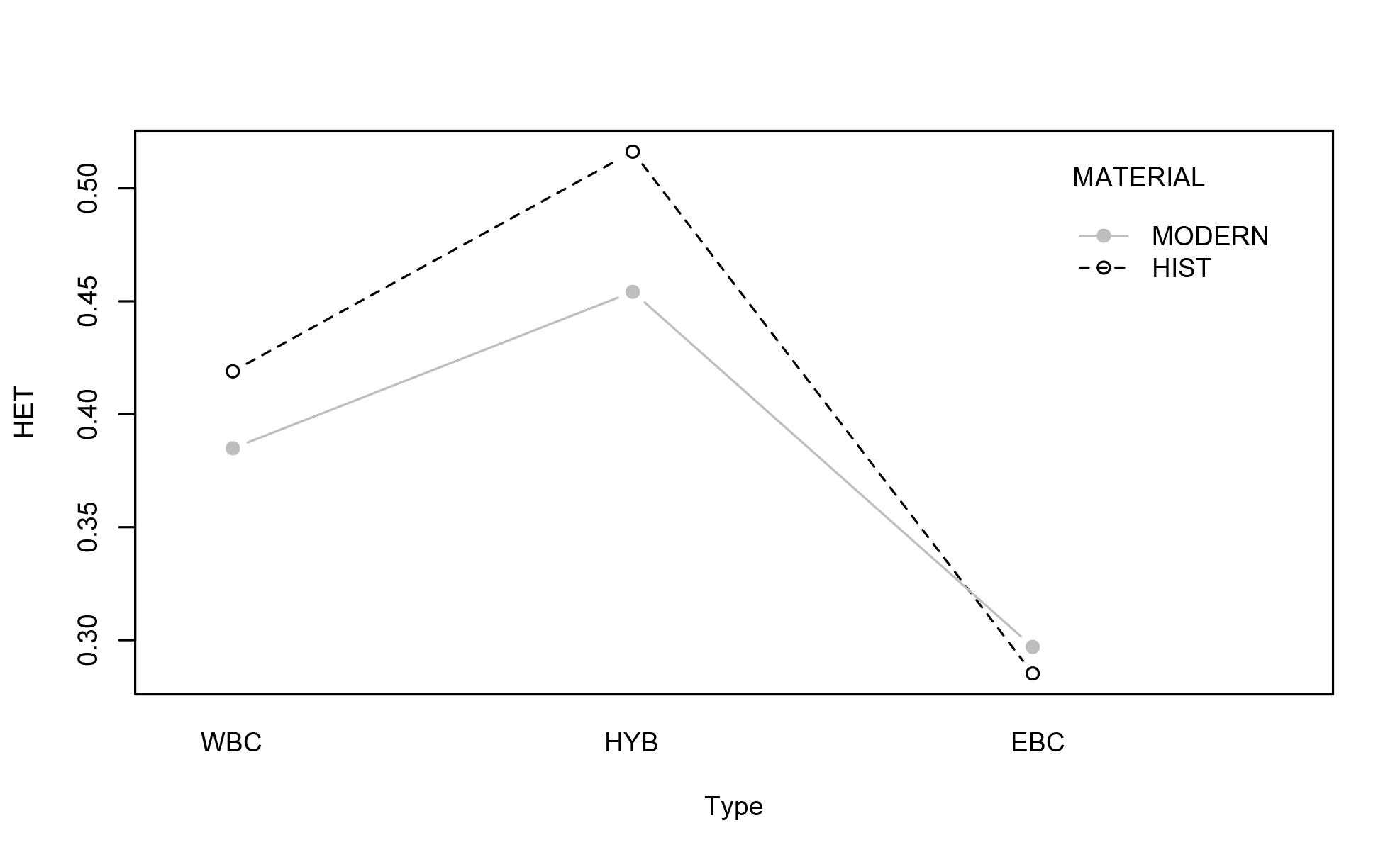


**Figure S13** Interaction plot for Two-way ANOVA for heterozygosity in 20 SNP dataset.

**3.1 Population structure and hybrid assessment**


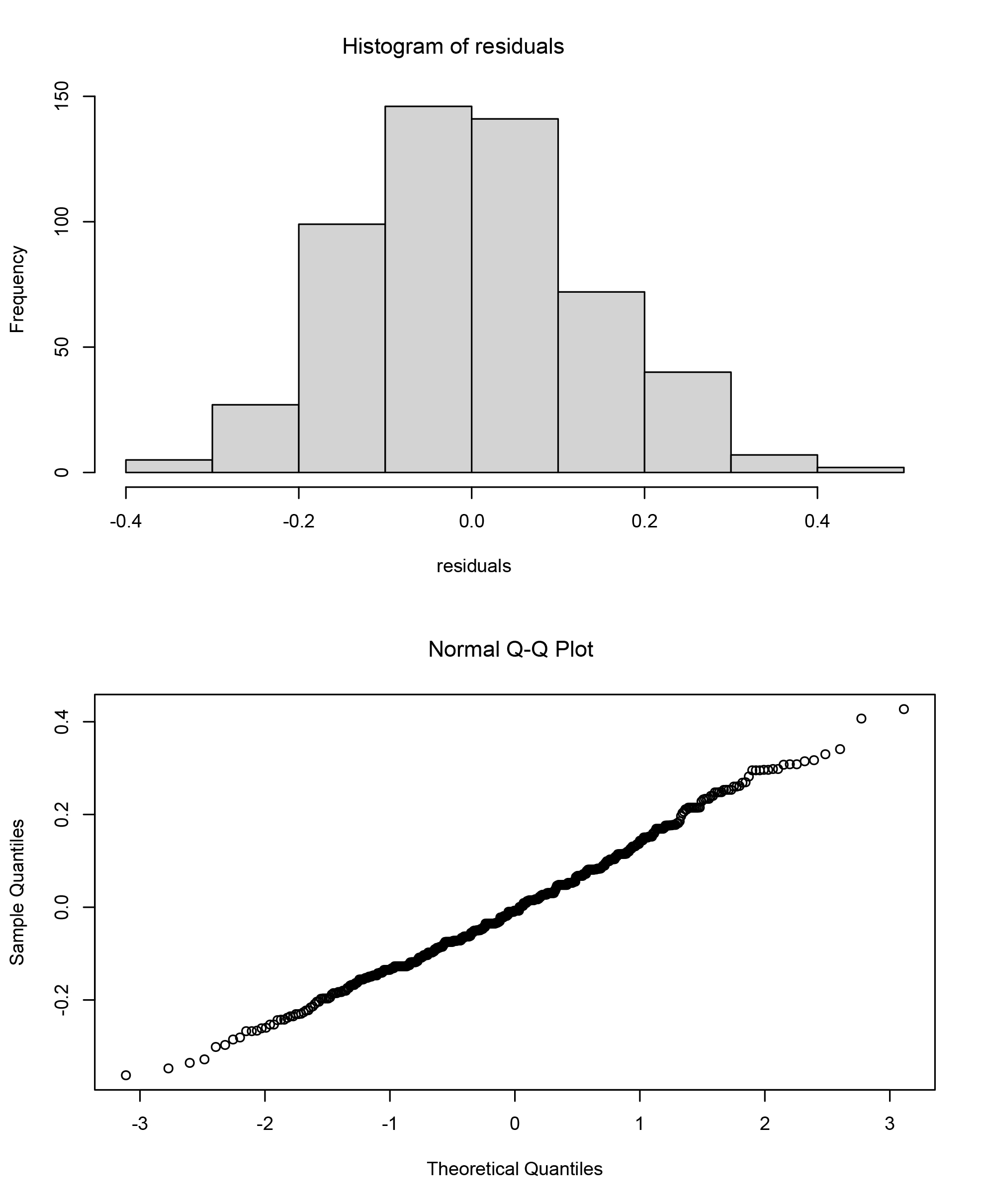


**B**

**A**

**Figure S14** Residual plots for Two-way ANOVA for heterozygosity in 20 SNP dataset. **A)** Histogram of residuals **B)** Q-Q-plot of the ANOVA.

**3.1 Population structure and hybrid assessment**

**Table S22** Post-hoc test statistics for Dunn test (Dunn, 1964) for Kruskal Wallis ANOVA. p-values adjusted according to the Benjamini-Hochberg method.

| **Dunn test for Kruskal Wallis ANOVA** | | | |
| --- | --- | --- | --- |
| ***Comparison*** | ***Z*** | ***p.unadj*** | ***p.adj*** |
| **WBC vs HYB** | -3.406598 | 6.577788e-04 | 6.577788e-04 |
| **WBC vs EBC** | 6.692702 | 2.190863e-11 | 3.286294e-11 |
| **HYB vs EBC** | 11.044200 | 2.338420e-28 | 7.015260e-28 |

#### **3.2 Inversion frequencies**

**Table S23** Fisher´s exact test for difference between areas in inversion genotype proportions for Baltic cod genotypes.

| ***Chromosome*** ***Type*** | **LG02** | **LG07** |
| --- | --- | --- |
| **WBC** | 1.88e-02 | 5.97e-03 |
| **HYB** | 3.43e-07 | 2.37e-04 |
| **EBC** | 3.8e-03 | 0.78 |

**Table S24** Fisher´s exact test for difference between areas in inversion genotype proportions at LG02 for Baltic cod genotypes, pinpointing after Bonferroni correction which areas that differ in inversion genotype proportions.

| **LG02** |  |  |  |  |
| --- | --- | --- | --- | --- |
| ***Type***  ***Area comparison*** | **WBC** | **HYB** | **EBC** |  |
| **MEC vs ARK** | 1.88e-02 | 2.8e-03 | 0.12 |  |
|  |  |  |  |  |
|  |  |  |  |  |
| **ARK vs BOR** | NA | 3.1e-03 | 0.02 |  |
|  |  |  |  |  |
|  |  |  |  |  |
| **MEC vs BOR** | NA | 2.99e-08 | 9.7e-04 |  |
|  |  |  |  |  |
|  |  |  |  |  |

**Table S25** Fisher´s exact test for difference between areas in inversion genotype proportions at LG07 for Baltic cod genotypes, pinpointing after Bonferroni correction which areas that differ in inversion genotype proportions.

| **LG07** |  |  |  |  |
| --- | --- | --- | --- | --- |
| ***Type***  ***Area comparison*** | **WBC** | **HYB** | **EBC** |  |
| **MEC vs ARK** | 5.97e-03 | 0.06 | 0.78 |  |
|  |  |  |  |  |
|  |  |  |  |  |
| **ARK vs BOR** | NA | 0.02 |  |  |
|  |  |  |  |  |
|  |  |  |  |  |
| **MEC vs BOR** | NA | 3.71e-05 |  |  |
|  |  |  |  |  |
|  |  |  |  |  |

**3.2 Inversion frequencies**

**Table S26** Fisher´s exact test for difference between Baltic cod types in inversion genotype proportions.

| ***Chromosome*** | ***Type significance*** |
| --- | --- |
| **LG02** | 4.36e-06 |
| **LG07** | 4.8e-03 |

**Table S27** Fisher´s exact test for difference between Baltic cod types in inversion genotype proportions at LG02 and LG07, pinpointing after Bonferroni correction which types that differ in inversion genotype proportions.

| ***Chromosome*** ***Comparison*** | **LG02** | **LG12** |
| --- | --- | --- |
| **WBC vs HYB** | 1.26e-04 | 2.09e-03 |
| **HYB vs EBC** | 0.23 | 0.02 |
| **WBC vs EBC** | 2.23e-06 | 0.13 |

**3.3 Inversion scoring**

**Table S28** Table for Chi-square test to test deviation from Hardy Weinberg equilibrium for inversion genotypes at LG12 for the western Baltic cod (WBC), the hybrids (HYB) and the eastern Baltic cod (EBC). Genotypes given as REF (homozygous as in reference genome gadMor2), HET (heterozygous) and NON_REF (non reference homozygous genotype), also in allele frequency REF - referring to the reference allele and the NON_REF as the non reference allele.

| ***LG02*** | ***Count (O)*** | ***Geno freq*** | ***Allele freq*** | ***Expected Geno freq (HWE)*** | ***Expected Geno counts (E)*** | ***(O-E)^2*** | ***(O-E)^2/E*** | |  |
| --- | --- | --- | --- | --- | --- | --- | --- | --- | --- |
| ***WBC*** |  |  |  |  |  |  |  |  |  |
| **REF** | 14 | 0.24 | 0.49 | 0.24 | 14 | 0 | 0 |  |  |
| **HET** | 29 | 0.5 |  | 0.5 | 29 | 0 | 0 |  |  |
| **NON_REF** | 15 | 0.26 | 0.51 | 0.26 | 15 | 0 | 0 | ***df*** | 1 |
| **Tot** | **58** | **1** | **1** | **1** | **58** | *χ* ***2*** | **0** | ***p*** | **1** |
| ***HYB*** | ***Count (O)*** | ***Geno freq*** | ***Allele freq*** | ***Expected Geno freq (HWE)*** | ***Expected Geno counts (E)*** | ***(O-E)^2*** | ***(O-E)^2/E*** | |  |
| **REF** | 51 | 0.53 | 0.73 | 0.54 | 52 | 1 | 0.01 |  |  |
| **HET** | 39 | 0.41 |  | 0.39 | 37 | 2 | 0.06 |  |  |
| **NON_REF** | 6 | 0.06 | 0.27 | 0.07 | 7 | 1 | 0.09 | ***df*** | 1 |
| **Tot** | **96** | **1** | **1** | **1** | **96** | *χ* ***2*** | **0.16** | ***p*** | **0.69** |
| ***EBC*** | ***Count (O)*** | ***Geno freq*** | ***Allele freq*** | ***Expected Geno freq (HWE)*** | ***Expected Geno counts (E)*** | ***(O-E)^2*** | ***(O-E)^2/E*** | |  |
| **REF** | 101 | 0.46 | 0.71 | 0.5 | 111 | 97 | 0.87 |  |  |
| **HET** | 111 | 0.5 |  | 0.41 | 91 | 386 | 4.23 |  |  |
| **NON_REF** | 9 | 0.04 | 0.29 | 0.09 | 19 | 97 | 5.13 | ***df*** | 1 |
| **Tot** | **221** | **1** | **1** | **1** | **221** | *χ* ***2*** | **10.23** | ***p*** | **0.001** |

**3.3 Inversion scoring**

**Table S29** Table for Chi-square test to test deviation from Hardy Weinberg equilibrium for inversion genotypes at LG12 for the western Baltic cod (WBC), the hybrids (HYB) and the eastern Baltic cod (EBC). Genotypes given as REF (homozygous as in reference genome gadMor2), HET (heterozygous) and NON_REF (non reference homozygous genotype), also in allele frequency REF - referring to the reference allele and the NON_REF as the non reference allele.

| ***LG12*** | ***Count (O)*** | ***Geno freq*** | ***Allele freq*** | ***Expected Geno freq (HWE)*** | ***Expected Geno counts (E)*** | ***(O-E)^2*** | ***(O-E)^2/E*** | |  |
| --- | --- | --- | --- | --- | --- | --- | --- | --- | --- |
| ***WBC*** |  |  |  |  |  |  |  |  |  |
| **REF** | 10 | 0.13 | 0.39 | 0.15 | 12 | 4 | 0.31 |  |  |
| **HET** | 41 | 0.53 |  | 0.48 | 37 | 15 | 0.4 |  |  |
| **NON_REF** | 27 | 0.35 | 0.61 | 0.37 | 29 | 4 | 0.13 | ***df*** | 1 |
| **Tot** | **78** | **1** | **1** | **1** | **78** | *χ* ***2*** | **0.84** | ***p*** | **0.36** |
| ***HYB*** | ***Count (O)*** | ***Geno freq*** | ***Allele freq*** | ***Expected Geno freq (HWE)*** | ***Expected Geno counts (E)*** | ***(O-E)^2*** | ***(O-E)^2/E*** | |  |
| **REF** | 32 | 0.34 | 0.58 | 0.33 | 31 | 1 | 0.05 |  |  |
| **HET** | 43 | 0.46 |  | 0.49 | 45 | 6 | 0.13 |  |  |
| **NON_REF** | 18 | 0.19 | 0.42 | 0.18 | 17 | 1 | 0.09 | ***df*** | 1 |
| **Tot** | **93** | **1** | **1** | **1** | **93** | *χ* ***2*** | **0.27** | ***p*** | **0.6** |
| ***EBC*** | ***Count (O)*** | ***Geno freq*** | ***Allele freq*** | ***Expected Geno freq (HWE)*** | ***Expected Geno counts (E)*** | ***(O-E)^2*** | ***(O-E)^2/E*** | |  |
| **REF** | 42 | 0.2 | 0.48 | 0.23 | 49 | 54 | 1.09 |  |  |
| **HET** | 122 | 0.57 |  | 0.5 | 107 | 216 | 2.01 |  |  |
| **NON_REF** | 51 | 0.24 | 0.52 | 0.27 | 58 | 54 | 0.92 | ***df*** | 1 |
| **Tot** | **215** | **1** | **1** | **1** | **215** | *χ* ***2*** | **4.03** | ***p*** | **0.04** |


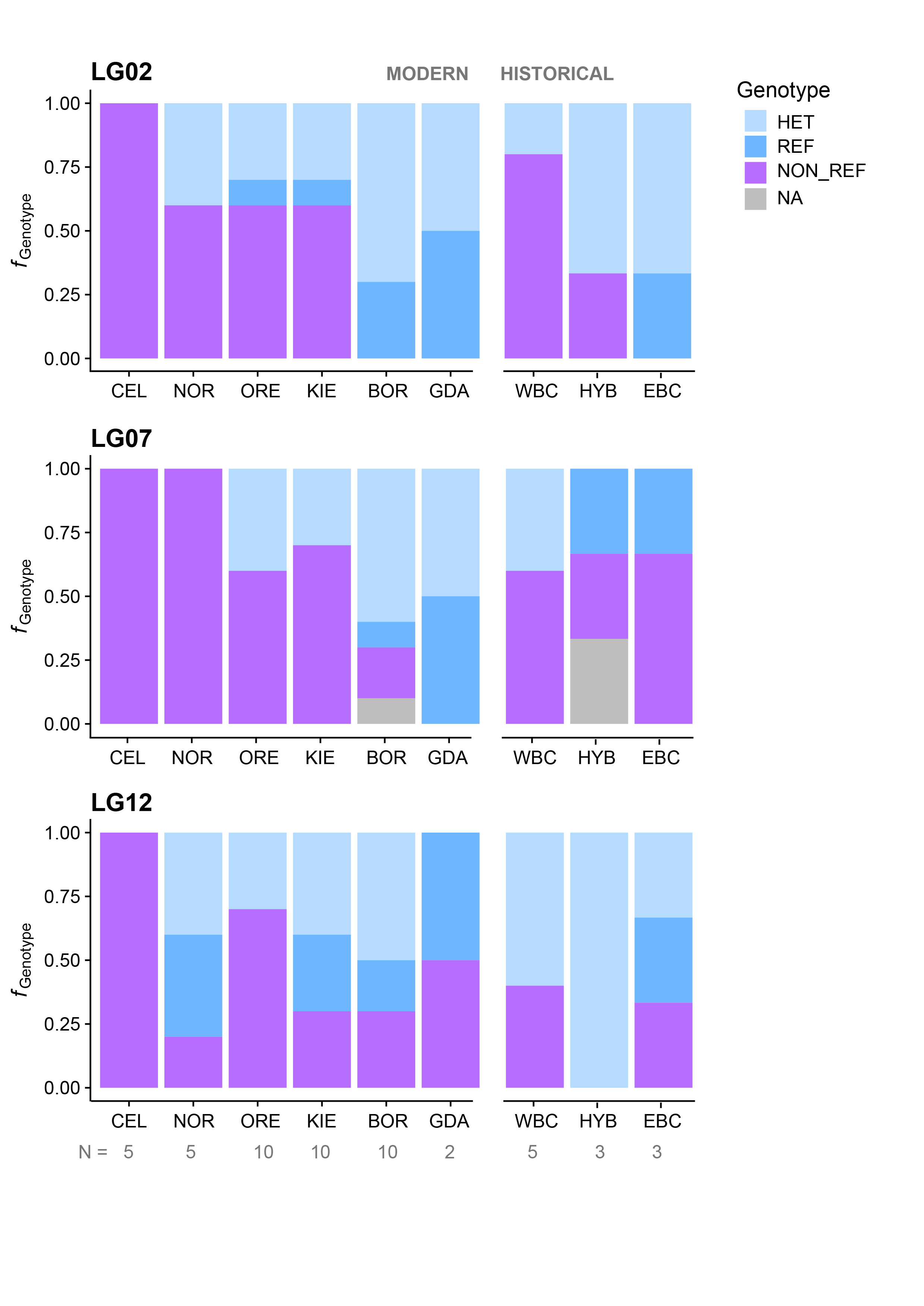


**Figure S15** Inversion plots showing f_Genotype_ (fraction of genotypes) of individuals in WGS dataset, showing modern populations from west to east and historical divided into western Baltic cod (WBC), hybrids (HYB) and eastern Baltic cod (EBC). Genotypes given as HET (heterozygous), REF (homozygous as in gadMor2), NON_REF (homozygous for non reference) and NA (non applicable, meaning genotype cannot be determined).

# **References**

CEES (2022). The Aqua Genome project. Available at: <https://www.aquagenome.uio.no> [Accessed April 3, 2022].

Dunn, O. J. (1964). Multiple Comparisons Using Rank Sums. *Technometrics* 6, 241–252. doi: 10.1080/00401706.1964.10490181.

Jónsson, H., Ginolhac, A., Schubert, M., Johnson, P. L. F., and Orlando, L. (2013). mapDamage2.0: fast approximate Bayesian estimates of ancient DNA damage parameters. *Bioinformatics* 29, 1682–1684. doi: 10.1093/bioinformatics/btt193.

Schubert, M., Ermini, L., Sarkissian, C. D., Jónsson, H., Ginolhac, A., Schaefer, R., et al. (2014). Characterization of ancient and modern genomes by SNP detection and phylogenomic and metagenomic analysis using PALEOMIX. *Nat Protoc* 9, 1056–1082. doi: 10.1038/nprot.2014.063.

Tomkiewicz, J., Tybjerg, L., and Jespersen, Å. (2003). Micro‐ and macroscopic characteristics to stage gonadal maturation of female Baltic cod. *J Fish Biol* 62, 253–275. doi: 10.1046/j.1095-8649.2003.00001.x.

Tørresen, O. K., Star, B., Jentoft, S., Reinar, W. B., Grove, H., Miller, J. R., et al. (2016). An improved genome assembly uncovers prolific tandem repeats in Atlantic cod. *Biorxiv*, 060921. doi: 10.1101/060921.

Weist, P., Schade, F. M., Damerau, M., Barth, J. M. I., Dierking, J., André, C., et al. (2019). Assessing SNP-markers to study population mixing and ecological adaptation in Baltic cod. *Plos One* 14, e0218127. doi: 10.1371/journal.pone.0218127.
